# Circular Polarimetric Imaging with a Metamaterial Integrated Long‐Wavelength Infrared Focal Plane Array

**DOI:** 10.1002/advs.202509292

**Published:** 2025-07-25

**Authors:** Tianyun Zhu, Ling Wang, Wenji Jing, Jie Deng, Jiexian Ye, Yujie Zhang, Zeshi Chu, Jing Zhou, Xiaoshuang Chen, Xiangyang Li, Wei Lu, Xuechu Shen

**Affiliations:** ^1^ State Key Laboratory of Infrared Physics Shanghai Institute of Technical Physics Chinese Academy of Sciences 500 Yu Tian Road Shanghai 200083 China; ^2^ University of Chinese Academy of Sciences 19 Yuquan Road Beijing 100049 China

**Keywords:** circular polarization detection, long‐wavelength infrared, one‐shot circular polarimetric imaging, quantum well infrared photodetector focal plane array, stokes parameter S_3_ sensing

## Abstract

Long‐wavelength infrared (LWIR) circular polarimetric imaging plays an important role in many areas. The immediacy of polarimetric imaging and the miniaturization of devices drive considerable efforts to division‐of‐focal‐plane‐array (DoFPA) circular polarimeters. However, the realization of such detectors is hampered by low polarization discrimination, reduced absorption in the detection material, and fabrication complexity. The situation becomes more serious in the LWIR range since the pixel size is only a few wavelengths of the incident light. Here, a quantum well infrared photodetector based LWIR DoFPA circular polarimeter featuring a 320 × 256 pixel array integrated with a chiral meta‐mirror array is established. The spectral range of this detector is from 10 to 11 µm. Taking advantage of the dual polarization selection, a CPER of 23.3 is achieved for the pixels integrated with the same chiral meta‐mirror structure, and a CPER of 5.67 for the pixels integrated with left‐ and right‐handed chiral meta‐mirror structures in a checkerboard pattern. The peak responsivity is improved by a factor of 9.13 compared to a standard reference device. With the LWIR DoFPA circular polarimeter, Stokes parameter *S*_3_ imaging is achieved with a noise equivalent *S*_3_ difference of 1.16×10^−4^, and demonstrate background suppression and target highlighting.

## Graphical abstract

A quantum well infrared photodetector based long‐wavelength infrared division‐of‐focal‐plane‐array circular polarimeter featuring a 320 × 256 pixel array integrated with a chiral meta‐mirror structure array is established. The device achieves a circular polarization extinction ratio of 5.67, a 9.13‐fold enhancement in responsivity, a noise equivalent *S*_3_ difference as small as 1.16 × 10^−4^, and enables background‐suppressed target‐highlighted imaging.

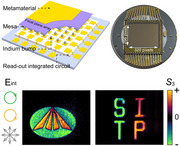

## 1 Introduction

Circular polarization detection has been widely used in circular dichroism spectroscopy,^[^ ^1^, ^2^, ^3^, ^4^ ^]^ vision dehazing,^[^ ^5^ ^]^ high‐fidelity communication,^[^ ^6^ ^]^ astronomical magnetic field sensing,^[^ ^7^ ^]^ optical encryption,^[^ ^8^, ^9^ ^]^ and biomolecular diagnosis.^[^ ^10^, ^11^, ^12^, ^13^ ^]^ Circular polarization detection discriminates left‐circularly polarized (LCP) light from right‐circularly polarized (RCP) light and provides the signal directly proportional to the Stokes parameter *S*_3_. Traditionally, like linear polarization detection, circular polarization detection is achieved by an optical system with discrete components, such as prisms, lenses, polarizers, and wave plates, in conjunction with a detector. These devices are broadly divided into two categories: “division of time” and “division of amplitude”. A division‐of‐time polarimeter acquires the intensities of different polarization states in a temporal sequence by rotating a waveplate or a polarizer, which severely limits the temporal resolution. A division‐of‐amplitude polarimeter splits the incident light into different photosensitive areas by a subtle optical system, which results in a large and complex structure. The immediacy of polarimetric imaging and the miniaturization of polarimeters drive considerable research efforts to division‐of‐focal‐plane‐array (DoFPA) devices, where the polarization discriminative photonic structures are directly integrated on the pixels.^[^ ^14^, ^15^, ^16^, ^17^ ^]^ This type of device can acquire the polarization information of the incident light in one shot and has the most compact structure.

However, the development of DoFPA polarimeters faces several challenges, such as rather low polarization extinction ratios (PERs), reduced light absorption in the detection material, and alignment issues during the integration of polarization discriminative structures. The PER is defined as a ratio of the responsivity of the primary detection polarization state to that of the polarization state orthogonal to the primary one. Due to light diffraction, scattering, and near field absorption at the pixels, the PER of a DoFPA polarimeter is 2 to 3 orders of magnitude lower than that of a polarimeter consisting of discrete polarization optics and a detector.^[^ ^18^, ^19^, ^20^, ^21^ ^]^ In the LWIR range, the situation becomes more serious, since the pixel size is only a few wavelengths of the incident light. The linear polarization extinction ratio (LPER) of an LWIR DoFPA polarimeter is typically below 5.^[^ ^22^ ^]^ In comparison, the circular polarization extinction ratio (CPER) of an integrated circular polarization detector is even lower,^[^ ^10^, ^23^, ^24^, ^25^, ^26^, ^27^ ^]^ since it is more difficult for a highly compact integrated structure to function like a circular polarizer, which should not only create a discriminative attenuation between two orthogonal polarization states but also control the phase retardation between these two polarization states. Light absorptance reduction is another important concern. Due to masking, scattering, and absorption by the integrated polarization discriminative structure, the absorptance of the detection material would be reduced by more than 50%. The sensitivity of LWIR detectors is quite limited compared with that of visible or near infrared detectors, and a reduction in responsivity of more than 50% is intolerable. Concerning the fabrication, most infrared FPAs are produced by bonding an infrared detection chip to a readout integrated circuit (ROIC) in a flip‐chip manner.^[^ ^28^, ^29^, ^30^ ^]^ Then, how to remove the substrate of the infrared detection chip and how to create an array of polarization discriminative structures that are well aligned with the detection pixels become technical difficulties.^[^ ^31^ ^]^ Due to the above issues, there are no reports of successful development of DoFPA circular polarimeters. In fact, these issues are common bottleneck problems in the development of optoelectronic metadevices.^[^ ^32^, ^33^, ^34^, ^35^, ^36^, ^37^, ^38^ ^]^

In this work, we address the three above challenges by photonic‐electronic codesign and large‐scale high‐precision alignment fabrication techniques, and establish a quantum well infrared photodetector (QWIP) based LWIR DoFPA circular polarimeter with 320 × 256 pixels (30 µm pixel pitch). The circular polarization discrimination is created by a chiral meta‐mirror structure that efficiently couples the incident light in the primary detection circular polarization state (LCP or RCP) into a surface plasmon polariton (SPP) mode, and reflects the light in the other circular polarization state (RCP or LCP). We removed the 500‐µm‐thick substrate after the flip‐chip process and obtained a 1‐µm‐thick, 10‐mm‐sized flat infrared detection chip. We have also developed a perforation alignment process that enables precise alignment of the chiral meta‐mirror array to the detector pixel array with an accuracy of tens of nanometers. In the FPA, the pixels integrated with left‐handed chiral meta‐mirrors and those integrated with right‐handed chiral meta‐mirrors are arranged in a checkerboard pattern, allowing us to acquire signals nearly proportional to *S*_3_ in one shot. In the advantage of dual polarization selection by the chiral meta‐mirror structure and by the quantum wells (QWs), our circular polarization detector exhibits a high CPER. For a 6 × 6 array of pixels integrated with the same chiral meta‐mirror structure, a CPER as high as 23.3 is achieved. For the LWIR DoFPA circular polarimeter with the checkerboard chiral meta‐mirror array, although the CPER drops to 5.67 due to more severe diffraction and scattering from adjacent pixels, it still exceeds those of most infrared integrated circular polarization detectors^[^ ^23^, ^24^, ^39^, ^40^, ^41^, ^42^ ^]^ and is sufficient for high‐quality circular polarimetric imaging.^[^ ^43^ ^]^ Based on the light field enhancement of the SPP mode, the peak responsivity is 9.13 times that of a standard reference device, which is a 45° edge facet coupled QWIP based on the same detection material. The LWIR DoFPA circular polarimeter achieves a responsivity of 5.94 ×10^7^ V W^−1^, a detectivity of 4.26 ×10^10^ Jones, a noise equivalent temperature difference (NETD) of 32 mK, and a noise equivalent *S*_3_ difference (NE*S*_3_D) of 1.16 × 10^−4^. This device demonstrates circular polarimetric imaging, capable of simultaneously detecting LCP and RCP light while suppressing background interference. Due to the inherent narrow spectral range of QWIPs and resonance based circular polarization discrimination of the chiral meta‐mirror, the spectral range (10–11 µm) of our device is not broad. In order for broader spectrum sensitivity and polarization discrimination, a discussion about utilizing a multi‐resonance metamaterial is included. Our work opens up a new avenue for the advancement of high‐performance LWIR DoFPA circular polarimeters.

## 2 Result

**Figure 1:**
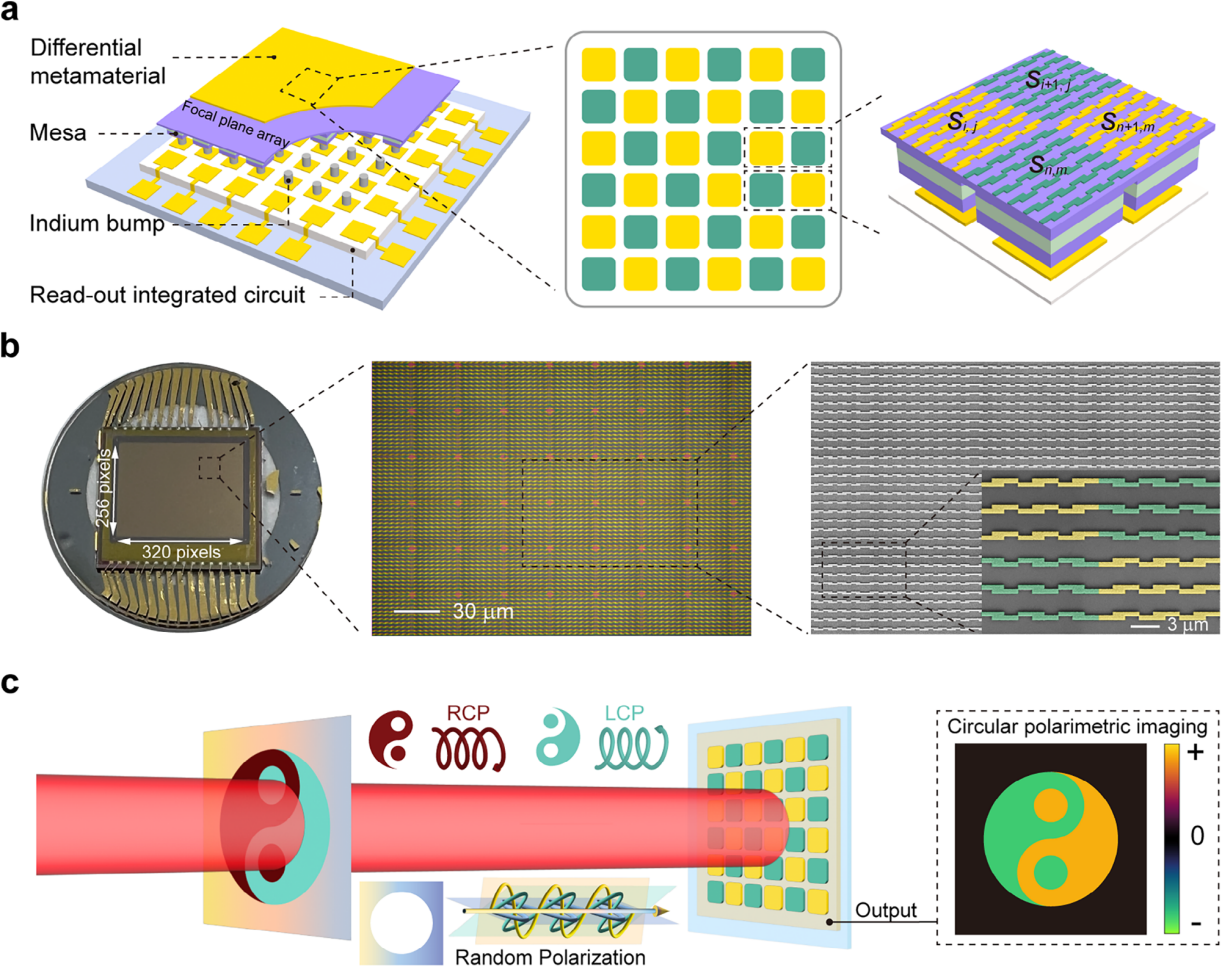
LWIR DoFPA circular polarimeter. a) Schematic diagram of the LWIR DoFPA circular polarimeter. One super pixel consists of 1 × 2 pixels, which are integrated with two chiral meta‐mirror structures of opposite chiralities, respectively. b) A photo picture of the LWIR DoFPA circular polarimeter chip (256 × 320 pixels). Micrograph of 48 pixels. SEM image of 8 pixels indicated in the micrograph. c) Schematic diagram of circular polarimetric imaging. The “yang” region (cyan) of the Taiji pattern is made of a circular polarizer transmitting LCP light, the “yin” region (deep red) is made of a circular polarizer transmitting RCP light, and the surrounding area on the polarization mask (gradient‐colored) does not change the polarization state of the incident light. The randomly polarized light passing through the mask is imaged as shown in the dashed frame on the right side.

The structure of the LWIR DoFPA circular polarimeter is schematically depicted in **Figure** **1**a. The detection material consists of 7 stacks of GaAs/AlGaAs QWs. The detailed structure of the detection material is described later. The chiral meta‐mirror array is arranged in a checkerboard pattern. One super pixel consists of 1 × 2 pixels, which are integrated with two chiral meta‐mirror structures of opposite chiralities, respectively. The circular polarimetric imaging pixel (*p*_*i*,*j*_) is calculated from the signals of the real pixels (*s*_*i*,*j*_). *i* denotes the column number and *j* the row number. *p*_*i*,*j*_ = *s*_*i*,*j*_ − *s*_*i* + 1, *j*_ when *i* and *j* are both odd or even. *p*_*i*,*j*_ = *s*_*i* + 1, *j*_ − *s*_*i*,*j*_ when one of *i* and *j* is an odd number and the other is even. If *i* is the last column, *p*_*i*,*j*_ takes the value of *p*_*i* − 1, *j*_. In this way, the signal of *p*_*i*,*j*_ is approximately proportional to the Stokes parameter *S*_3_. The entire FPA chip is scaled to 320 × 256 pixels with a pixel pitch of 30 µm, as shown in Figure 1b. The FPA chip is bonded to a ROIC in a flip‐chip manner. Using precision mechanical grinding and chemical etching, we thinned the QW substrate by ≈500 µm, resulting in an infrared detection chip with a thickness of only ≈1 µm, a lateral size of 10 mm × 8 mm, and a smooth surface. In addition, we innovate a perforation alignment technique that allows us to create chiral meta‐mirrors on each pixel, achieving an alignment accuracy of tens of nanometers. The alignment marks were fabricated on the backside of the chip. After flip‐chip bonding, an etching process was employed to expose these alignment marks on the front side. The detailed fabrication process is described in Note S1 (Supporting Information). The circular polarimetric imaging principle of our device is illustrated in Figure 1c. A randomly polarized light beam passes through a polarization mask with a Taiji pattern, where the “yang” region is made into a circular polarizer to transmit LCP light, the “yin” region is made into a circular polarizer to transmit RCP light, and the surrounding area does not change the polarization state of the light beam. Then, a Taiji pattern with a dark surrounding area is expected when the light beam reaches our device. The LCP light induces positive photoresponse, the RCP light induces negative photoresponse, and the randomly polarized light induces zero photoresponse. According to our previous study,^[^ ^43^ ^]^ the photoresponse is a dot product of the Stokes vector of the incident light ($S=[\begin{array}{cccc} S_{0} & S_{1} & S_{2} & S_{3} \end{array}]$) and the optoelectronic polarization eigen vector (OPEV $\mu =[\begin{array}{cccc} \mu_{0} & \mu_{1} & \mu_{2} & \mu_{3} \end{array}]$), i.e., *I*_ph_ = S ∙ µ. Then, the OPEV of *p*_*i*,*j*_ is evaluated to be $\left[\begin{array}{cccc} 0 & 0 & 2\mu_{2} & 2\mu_{3} \end{array}\right]$, where *µ*_2_ = −0.2 A W^−1^ and *µ*_3_ = 1.65 A W^−1^ at the peak wavelength, indicating that the signal of *p*_*i*,*j*_ is zero when the incident light is randomly polarized, horizontally polarized, or vertically polarized, and the signal for 45° or 135° linearly polarized light is much smaller than that for LCP or RCP light. Consequently, the signal of *p*_*i*,*j*_ can be regarded as almost proportional to *S*_3_, thereby allowing circular polarimetric imaging. Detailed information is given in Note S2 (Supporting Information).

**Figure 2:**
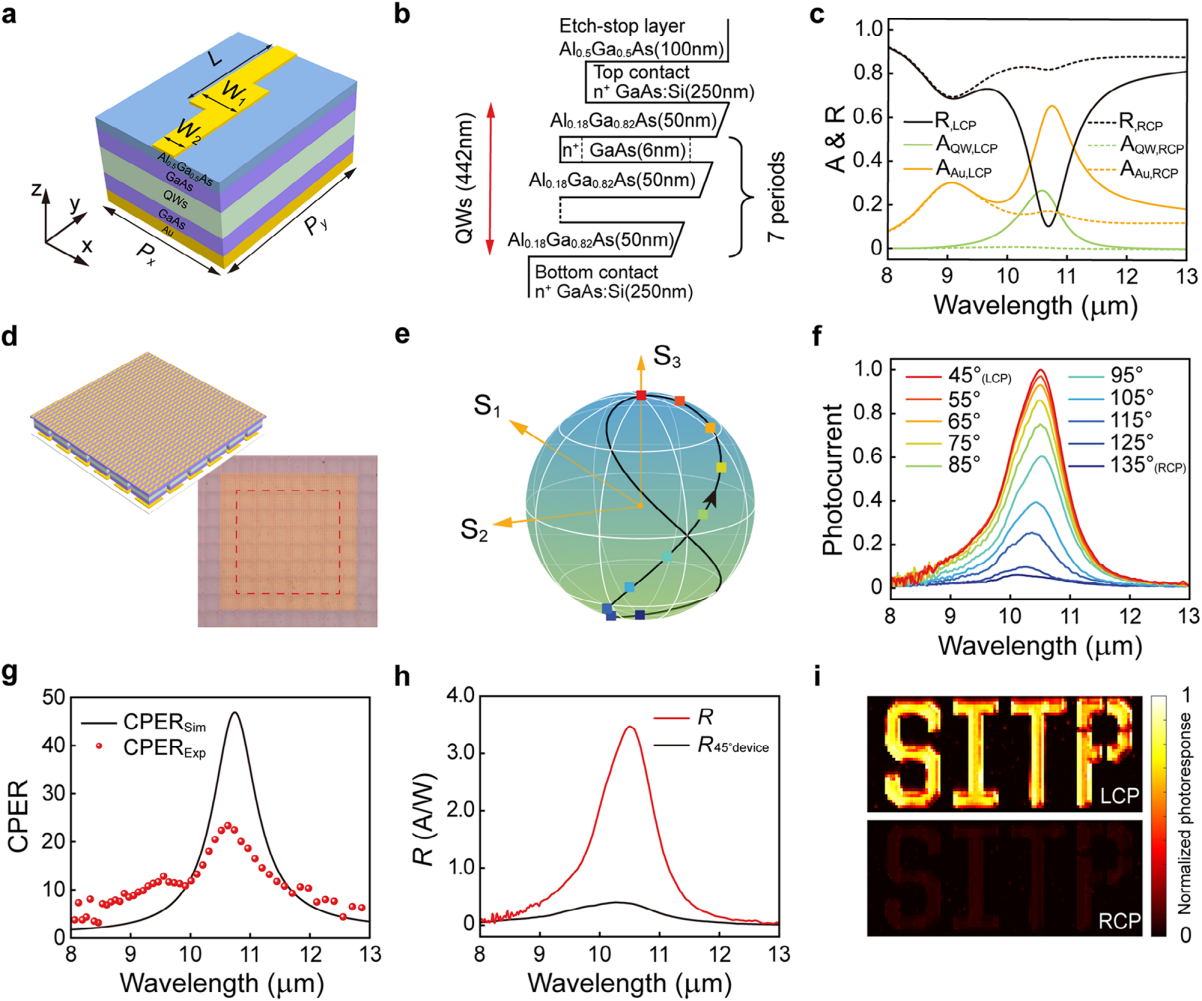
Optoelectronic properties of a QWIP integrated with the left‐handed chiral meta‐mirror. a) Schematic diagram of a single period of the left‐handed chiral meta‐mirror integrated QWIP. *P_x_* = 2500 nm, *P_y_* = 3750 nm, *W*_1_ = 783 nm, *W*_2_ = 431 nm, *L* = 2437 nm. b) Band diagram of the QWs. The thickness of each epitaxial layer is indicated. c) Simulated spectra of reflectance (R), QW absorptance (A_QW_), and Au absorptance (A_Au_) of the left‐handed chiral meta‐mirror integrated QWIP under LCP and RCP illumination. d) Schematic and optical photograph of the left‐handed chiral meta‐mirror integrated QWIP consisting of a 6 × 6 array of pixels. Electrical contacts are fabricated exclusively on the 6 × 6 pixels enclosed by the red dashed box for signal extraction. e) Polarization states on the Poincaré sphere corresponding to different QWP angles. The black line represents the trajectory of polarization state varying with the rotating QWP. f) Photocurrent spectra at different QWP angles. 45° and 135° correspond to RCP and LCP light, respectively. g) Wavelength dependent CPER obtained by simulation (solid line) and experiment (red dots). h) Responsivity spectra of the left‐handed chiral meta‐mirror integrated QWIP and the 45° edge facet coupled QWIP made of the same detection material under LCP illumination. i) Mask scanning imaging with the left‐handed chiral meta‐mirror integrated QWIP under LCP and RCP illumination.

In each pixel, the chiral meta‐mirror structure is in situ integrated with the detection material. The chiral meta‐mirror consists of an Au reflector at the bottom and a *Z*‐shaped antenna at the top. The *Z*‐shaped antenna is a periodic structure defined by five geometric parameters, *P_x_*, *P_y_*, *W*_1_, *W*_2_, and *L*, as shown in **Figure** **2**a, and it is made of a 50 nm thick metal layer (3 nm titanium and 47 nm gold). In our device, *P_x_* = 2500 nm, *P_y_* = 3750 nm, *W*_1_ = 783 nm, *W*_2_ = 431 nm, and *L* = 2437 nm. The detection material is sandwiched between the bottom Au reflector and the *Z*‐shaped antenna layer, and consists of 7 stacks of AlGaAs (50 nm)/GaAs (6 nm) QWs, two 250 nm thick heavily doped GaAs contact layers, and a 150 nm thick etch stop layer (Al_0.5_Ga_0.5_As). The electronic band diagram of the detection material is shown in Figure 2b. Due to the selection rule of the intersubband transition, the QWs can only absorb the light with a *z*‐component electric field (*E_z_*). Consequently, in the simulation, the QWs are modeled as a uniaxial medium with a diagonal dielectric constant tensor: *ε* = diag (*ε_x_*, *ε_y_*, *ε_z_*). Along the *x*‐ or *y*‐direction, the medium is a dielectric with the relative permittivity *ε_x_* = *ε_y_* = *ε_GaAs_*. In the *z*‐direction, the intersubband transition is described by a Lorentz oscillator $\varepsilon_{z}=\varepsilon_{GaAs}+\varepsilon_{GaAs}f_{12}\omega_{p}^{2}/\left(\omega_{0}^{2}-\omega^{2}-i\gamma \omega \right)$.^[^ ^44^, ^45^ ^]^ The optical properties of the QWs are detailed in Note S3 (Supporting Information). We use COMSOL Multiphysics to simulate the reflection and absorption spectra of the chiral meta‐mirror integrated QWIP. The absorption spectra include the absorptance of the QWs and the absorptance of the metallic parts. As shown in Figure 2c, for the left‐handed chiral meta‐mirror integrated QWIP, LCP light apparently excites a resonance around the wavelength of 10.6 µm. This resonance corresponds to the excitation of the SPP mode, which significantly enhances *E_z_* at the QWs, leading to an increase in the absorptance of the QWs (26.7%). In contrast, RCP light is highly reflected, resulting in a very low absorptance of the QWs (≈ 0.6%). Then, the CPER reaches 42 at 10.6 µm and achieves its maximum value of 46 at 10.75 µm. More details about the impact of the 3‐nm‐thick titanium on simulation results are provided in Note S4 (Supporting Information). In the context of a QWIP FPA, the pixel pitch is 30 µm, and the mesa size is 27 × 27 µm2. Thus, each mesa contains 8 × 12 periods of *Z*‐shaped antenna. Figure 2d shows a detector consisting of a 6 × 6 array of pixels connected in parallel, where all pixels are integrated with the same left‐handed chiral meta‐mirror. The photoresponse spectra for different polarization states are experimentally characterized by a set of circular polarization optics and a Fourier transform infrared (FTIR) spectrometer. The set of circular polarization optics contains a broadband linear polarizer and a broadband quarter‐wave plate (QWP). The infrared light from the FTIR spectrometer passes through the polarizer and the quarter‐wave plate in sequence. More details about the characterization setup are provided in Note S5 (Supporting Information). By rotating the quarter‐wave plate, the incident light can be gradually changed from RCP to LCP. The trajectory of the polarization state change is indicated by a black line on the Poincaré sphere in Figure 2e. The square dots on the trajectory correspond to the photocurrent spectra in Figure 2f. As the angle of the quarter‐wave plate increases from 45° (RCP) to 135° (LCP), the LCP component of the incident light gradually increases, leading to an increase in the photoresponse. The maximum CPER reaches 23.3 at 10.5 µm (Figure 2g). This record‐high CPER for integrated circular polarization detectors is attributed to the dual polarization selection mechanism, which is described in detail in the next section. The photocurrent spectra at different QWP angles (Figure 2f) are used to calculate the wavelength dependent OPEV.

Moreover, the SPP resonance excited by the LCP light results in a high optical coupling efficiency and thus a high peak responsivity of 3.47 A W^−1^, which is 9.13 times that of a standard reference device, i.e., a 45° edge facet coupled QWIP (Details in Note S6, Supporting Information). A mask‐scanning single‐detector imaging experiment was performed to demonstrate the circular polarization discrimination ability of the left‐handed chiral meta‐mirror integrated detector. The mask is made of a piece of steel with a perforated pattern “SITP”, and it is inserted in the light path. The incident light is generated by a CO_2_ laser with a wavelength of 10.6 µm and is set to LCP light. The image is generated by recording the photocurrent of our detector as the laser spot scans the mask. As shown in Figure 2i, the distinction between LCP and RCP light during the imaging by the chiral meta‐mirror integrated pixel is prominent. See Note S5 (Supporting Information) for more details about this experiment.

## 3 Analysis

**Figure 3:**
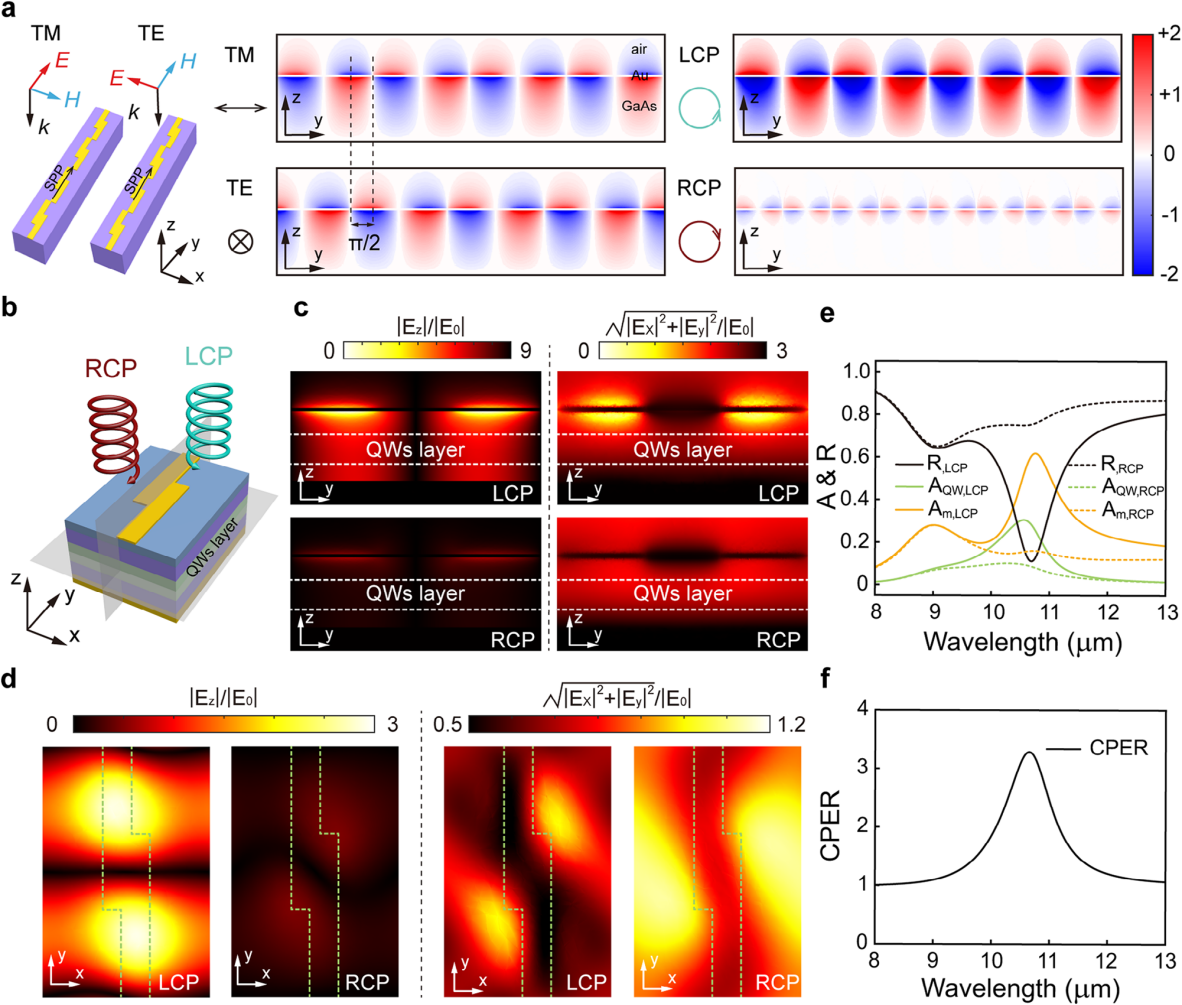
High CPER induced by the double polarization selection mechanism. a) Schematic illustration of the constructive and destructive interference between the SPPs excited by the *E_x_* and *E_y_* field of LCP and RCP light. b) Schematic of one period of chiral meta‐mirror integrated QWIP and two cross‐sections. The *y‐z* cross‐section is at the center of this one period structure, and the *x‐y* cross‐section is at the center of the QWs layer. c, d) Distributions of |*E_z_* |/|*E*_0_| and $\sqrt{{|E_{x}|}^{2}+{|E_{y}|}^{2}}/\left|E_{0}\right|$ in the *y‐z* cross‐section c) and in the *x‐y* cross‐section d) under LCP and RCP illumination. e) Simulated spectra of reflectance (R), QW absorptance (A_QW_), and Au absorptance (A_Au_) of the chiral meta‐mirror integrated isotropic QWIP under LCP and RCP illumination. f) Wavelength dependent CPER of the chiral meta‐mirror integrated isotropic QWIP.

The high CPER of the chiral meta‐mirror integrated QWIP originates from a dual polarization selection mechanism. The dual polarization selection includes the first polarization selection by the chiral meta‐mirror and the second polarization selection by the anisotropic absorption of the QWs. The circular polarization selectivity provided by the chiral meta‐mirror is attributed to the constructive and destructive interference between SPPs excited by the *E_x_* and *E_y_* components of the incident light field, as shown in **Figure** **3**a. Due to the periodic structure of the *Z*‐shaped antennas along the *y*‐direction, the incident light can excite SPP propagating along the *y*‐direction at the interface between the *Z*‐shaped antennas and the detection material. Typically, SPP can only be excited by TM waves (*E_y_*) and not by TE waves (*E_x_*). However, due to the twisted structure of the *Z*‐shaped antennas, the *E_x_* component of the TE wave simultaneously generates both the polarization‐unconverted scattered field and the polarization‐converted scattered field (*E_y_* component), thereby exciting the SPP. Circularly polarized light can be considered as the coherent superposition of TM and TE waves. For LCP light, the *E_x_* component leads the *E_y_* component by π/2; for RCP light, the *E_x_* component lags the *E_y_* component by π/2. Therefore, when the phase difference between the SPP excited by *E_x_* and that excited by *E_y_* is −π/2, the two SPP waves excited by the *E_x_* and *E_y_* components of LCP light interfere constructively. In contrast, the two SPP waves excited by the *E_x_* and *E_y_* components of RCP light interfere destructively.^[^ ^46^ ^]^ Consequently, LCP light effectively excites the SPP mode, whereas RCP light does not. The origin of circular polarization selectivity in the chiral meta‐mirror integrated structure can be quantitatively analyzed through the amplitude and phase of the reflected fields. Detailed analysis is provided in Note S7 (Supporting Information).

The second polarization selection is caused by the interaction between the light field and the QWs. Due to the selection rule of the intersubband transition of the QWs, it can only be excited by light with the *E_z_* component. The absorption of the QWs writes:

(1) $$A_{QW}=\underset{QW}{\;\int \int \int \;}\frac{1}{2}\omega Im\left(\varepsilon_{z}\right){\left|E_{z}\right|}^{2}dV$$

Figure 3c,d present the distributions of |*E*_z_|/|*E*_0_| and $\sqrt{{|E_{x}|}^{2}+{|E_{y}|}^{2}}/\left|E_{0}\right|$ on the two cross‐sections marked in Figure 3b. *E*_0_ is the electric field of the incident light. For LCP light, the SPP mode is excited at the interface between the top metallic metamaterial and the QWs material at 10.6 µm. The primary polarization direction of the SPP wave (*z* direction) is aligned with the absorption direction of the QWs, leading to a significant enhancement in QWs absorption, as illustrated in Figure 3c,d. Within the QWs layer, the *z* component of the electric field energy accounts for 88% of the total electric field energy (|*E_z_* |^2^/(|*E_x_* |^2^ + |*E_y_* |^2^ + |*E_z_* |^2^)). For RCP light, most of the incident light is reflected, and the light entering the QWs layer is predominantly polarized in the *x* or *y* direction. The energy in the *z*‐direction is ≈6.7% of the total energy, which results in suppressed absorption in the QWs material.

The combined effect of polarization selection by the chiral meta‐mirror and the anisotropic active material results in a high CPER. For comparison, we performed a numerical study of the case where the QWs become an isotropic detection material, i.e., *ε_x_* = *ε_y_* = *ε_z_*. The reflection and absorption spectra of the chiral meta‐mirror integrated isotropic QWIP for LCP and RCP light are presented in Figure 3e. Compared with the absorption spectra of the chiral meta‐mirror integrated anisotropic QWIP (Figure 2c), for LCP light, the absorptance of the QWs at the wavelength of 10.6 µm increases only slightly from 26.7% to 30.3%, since the electric field energy of the SPP is primarily distributed in the *z* direction. In contrast, for RCP light, the absorptance of the QWs increases significantly from 0.6% to 9.3%, since the considerable *E_x_* and *E_y_* can also induce absorption in the isotropic QWs. As a result, the peak CPER diminishes drastically to ≈3 due to the breakdown of the dual‐polarization selection mechanism, as shown in Figure 3f, which is comparable to the performance of other chiral metamaterial integrated detectors.^[^ ^25^, ^27^, ^39^, ^40^, ^41^, ^42^ ^]^

## 4 Optoelectronic Characteristics of the LWIR DoFPA Circular Polarimeter

**Figure 4:**
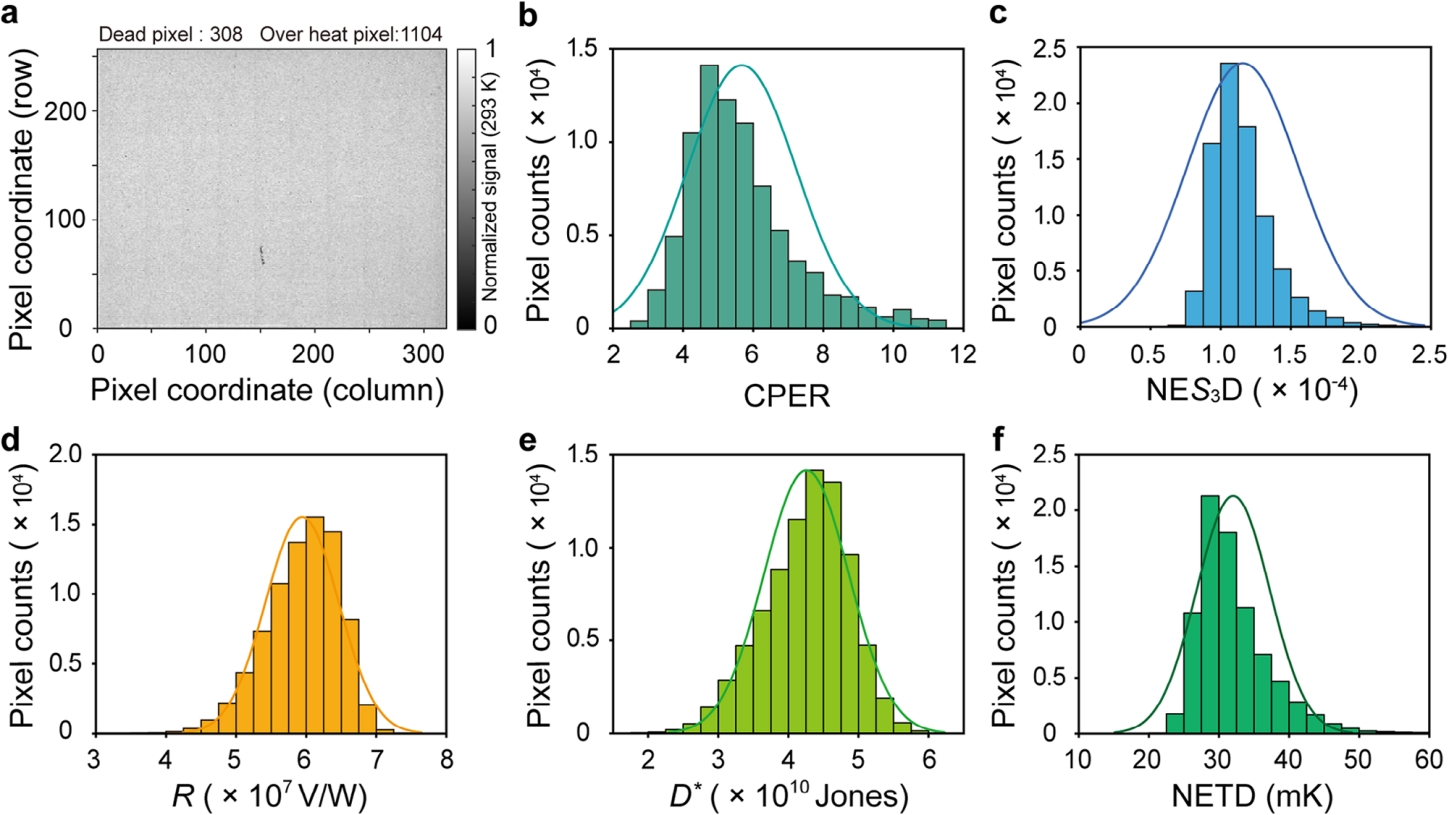
Optoelectronic characteristics of the LWIR DoFPA circular polarimeter. a) Normalized signal distribution of the LWIR DoFPA circular polarimeter illuminated by a 293 K surface‐emitting blackbody. b) Histogram of the CPER at the wavelength of 10.6 µm for all the pixels of the LWIR DoFPA circular polarimeter. c) Histogram of the noise equivalent *S*_3_ difference (NE*S*_3_D). d) Histogram of the responsivity (*R*). e) Histogram of the detectivity (D^*^). f) Histogram of the noise equivalent temperature difference (NETD). In b)‐f), the vertical axis represents the number of pixels.

The normalized signal distribution at 293 K is exhibited in **Figure** **4**a. The operable pixel factor is 98.3% and the photoresponse non‐uniformity is 8.8% without correction. The chiral meta‐mirror array is arranged in a checkerboard pattern to achieve in situ measurement of the Stokes parameter *S*_3_. However, this arrangement reduces the CPER for each pixel due to more severe diffraction and scattering from adjacent pixels. As shown in Figure 4b, the CPER distribution for the checkerboard QWIP FPA has a mean value of 5.67. Although the CPER of the checkerboard FPA is lower than that of the (6 × 6)‐pixel detector, where all the pixels are integrated with the same chiral meta‐mirror, a CPER of 5.67 is sufficient for high accuracy circular polarimetric imaging,^[^ ^43^ ^]^ and it remains the highest among all reported integrated infrared circular polarization detectors. The measurement results show that the noise equivalent *S*_3_ difference (NE*S*_3_D) distribution of our FPA under blackbody irradiation has an average value of 1.16 × 10^−4^ (Figure 4c). The calculation details for NE*S*_3_D are provided in Note S8 (Supporting Information).

After completing the flip‐chip bonding process for the QWIP FPA, we performed in situ integration of the chiral meta‐mirrors through a series of processes including substrate removal, perforation etching, e‐beam lithography, metal deposition, and lift‐off. Despite these additional steps, the QWIP FPA maintains high performance. As shown in Figure 4d–f, at a bias voltage of −0.7 V and an operating temperature of 45 K, the average responsivity of QWIP FPA is 5.94 × 10^7^ V W^−1^, the detectivity is 4.26 × 10^10^ Jones, and the NETD is 32 mK. Performance metrics of the QWIP FPA at varying operating temperatures are provided in Note S9 (Supporting Information).

**Figure 5:**
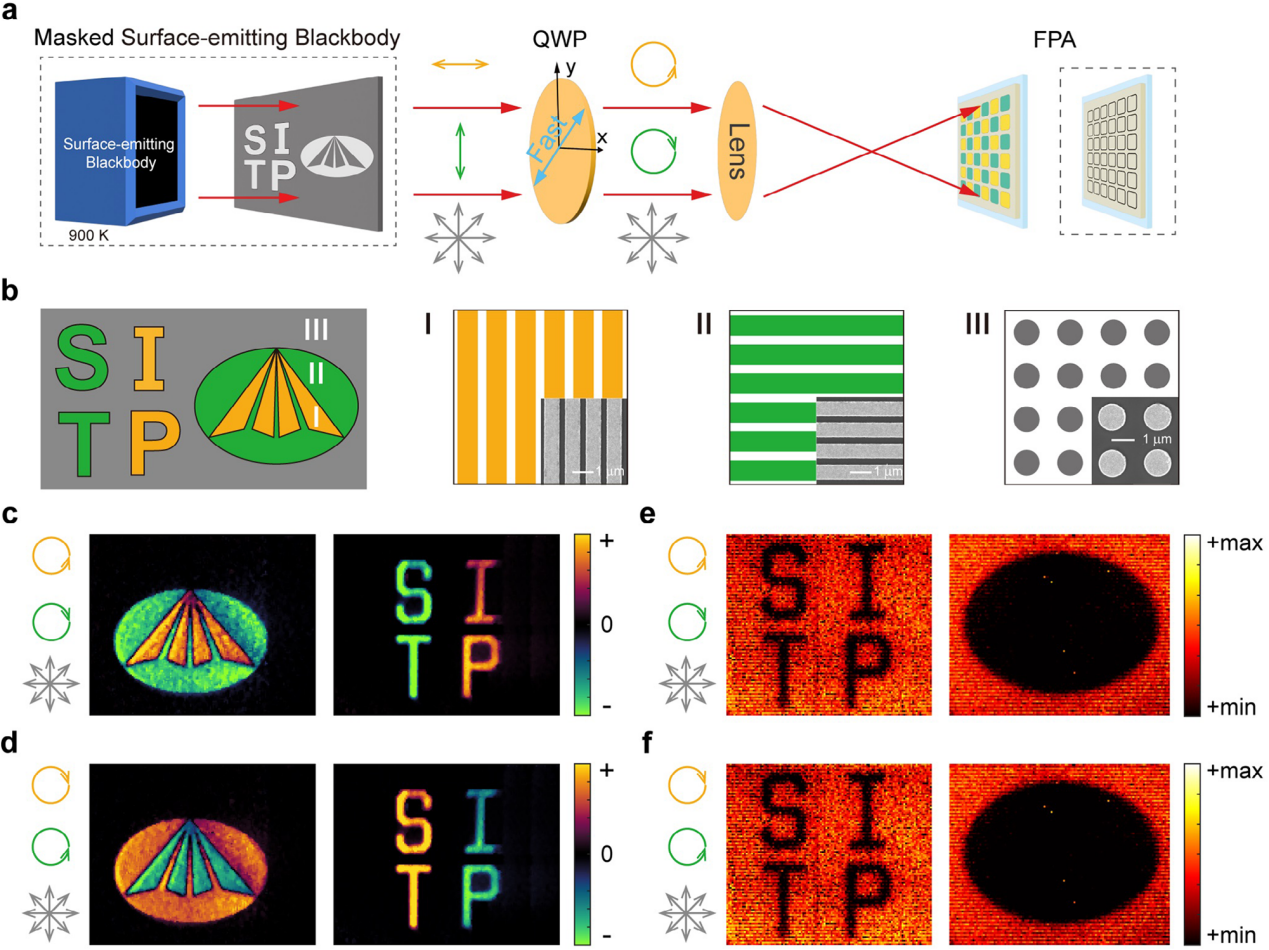
Circular polarimetric imaging with the LWIR DoFPA circular polarimeter. a) Schematic illustration of the experimental setup for circular polarimetric imaging. The device in the dashed frame on the right‐hand side represents an ordinary FPA without chiral meta‐mirror structures. b) Schematic of the polarization mask. The Type‐I regions on the mask function as horizontal polarizers. The Type‐II regions function as vertical polarizers. The Type‐III regions do not change the polarization state of the incident light. Adjacent on the right are magnified structural schematics and SEM images for these regions. c, d) Circular polarimetric imaging results using the LWIR DoFPA circular polarimeter when the fast axis of the quarter‐wave plate is at a 45° angle or a 135° angle to the positive *x*‐axis. e, f) Imaging results using an ordinary QWIP FPA when the fast axis of the quarter‐wave plate is at a 45° angle or a 135° angle to the positive *x*‐axis.

With this LWIR DoFPA circular polarimeter, we performed circular polarimetric imaging experiments. The imaging set up is illustrated in **Figure** **5**a. The target is a masked surface‐emitting blackbody with a temperature of 900 K. The thermal radiation of the surface‐emitting blackbody is randomly polarized, and its polarization state is changed by the mask and a quarter wave plate. As the thermal radiation passes through an imaging lens, the pattern on the mask is projected on the LWIR DoFPA circular polarimeter. The mask is a Si chip covered with three types of metal structures: I) vertical metal strip array, II) horizontal metal strip array, III) metal disk array, as shown in Figure 5b. The metal structures are fabricated by deep ultraviolet photolithography and deposition of 10 nm Ti/90 nm Au. Type‐I regions transmit horizontally polarized light. Type‐II regions transmit vertically polarized light. Type‐III regions do not change the polarization state of the transmitted light. The triangular areas and the areas of the two letters “I” and “P” belong to Type‐I regions. The ellipse area and the areas of the two letters “S” and “T” belong to Type‐II regions. The rest areas on the mask transmit randomly polarized light. A quarter‐wave plate is introduced after the mask to generate circularly polarized light. The fast axis of this quarter‐wave plate is oriented at 45° to the positive *x*‐axis. Then, the *x*‐polarized light from Type‐I regions is converted into LCP light, the *y*‐polarized light from Type‐II regions is converted into RCP light, and the randomly polarized light from Type‐III regions remains randomly polarized. Further details can be found in Note S10 (Supporting Information). As shown in Figure 5c, the LWIR DoFPA circular polarimeter clearly shows the pattern of the mask. The image signal provided by the polarimeter is proportional to *S*_3_. The signal of LCP light is positive and that of RCP light is negative. The signal of randomly polarized light is zero, so the background interference is suppressed. When the fast axis of the quarter‐wave plate is oriented at 135° to the positive direction of the *x*‐axis, the light from Type‐I regions becomes RCP light, the light from Type‐II regions becomes LCP light, and the light from Type‐III regions remains randomly polarized. As a result, the polarity of the circular polarimetric imaging (Figure 5d) is opposite to that in Figure 5c. For comparison, a QWIP FPA without the chiral meta‐mirror array is also employed to image the target. The ordinary FPA is able to identify differences in intensity. Since the Type‐I and Type‐II regions on the mask work as wire grid polarizers, half the power is lost when the randomly polarized thermal radiation passes through these regions. Thus, the signals from the Type‐I and Type‐II regions are weaker than those from the Type‐III regions. As a result, we can observe the letters “SITP” and the ellipse in Figure 5e,f. In this case, the background is not suppressed but becomes stronger than the targets. Once there is a disturbance in the high background, the detection of the targets would be severely interfered. The ordinary FPA cannot discriminate LCP and RCP light and cannot capture the signal of *S*_3_. As a result, the triangles inside the ellipse disappear, since the signals from the Type‐I regions and those from the Type‐II regions have the same polarity and intensity.

The intersubband transition governed by strict selection rules of the QWIP and resonance based circular polarization discrimination of the chiral meta‐mirror results in a limited spectral range (10–11 µm) of our device. However, broad‐spectrum sensitivity with polarization discrimination is essential for many applications. To overcome the narrow‐band problem, we propose a multi‐resonance chiral meta‐mirror structure, which combines three chiral meta‐mirror structures with three different resonances. It is numerically demonstrated that the multi‐resonance metamaterial integrated QWIP has a peak absorptance similar to that of the device presented above, and a spectral range (2 µm) being doubled. More details are provided in Note S11 (Supporting Information).

## 5 Conclusion

In this work, we establish a LWIR DoFPA circular polarimeter by addressing the challenges of low polarization discrimination, reduced absorption of the detection material and alignment complexity during the fabrication process. This circular polarimeter is based on a QWIP FPA with 320 × 256 pixels (pixel pitch equal to 30 µm). The pixel array is in situ integrated with a chiral meta‐mirror array. In the FPA, half of the pixels are integrated with left‐handed chiral meta‐mirrors, while the other half are integrated with right‐handed chiral meta‐mirrors, arranged in a checkerboard pattern. This configuration allows for near in situ sensing of the Stokes parameter *S*_3_ in one shot. By leveraging the dual polarization selection mechanism, a CPER as high as 23.3 is achieved for a 6 × 6 array of pixels, each integrated with the same chiral meta‐mirror structure. For the checkerboard layout of the chiral meta‐mirrors, the CPER decreases to 5.67, but still exceeds those of most infrared integrated circular polarization detectors and is sufficient for our device to achieve high‐quality circular polarimetric imaging. Moreover, the incident light with the primary detection circular polarization efficiently excites an SPP wave that enhances the absorption of the QWs, leading to a responsivity 9.13 times that of a standard reference device, which is a 45° edge facet coupled QWIP based on the same detection material. Concerning the fabrication, we have developed modified mechanical grinding and chemical etching processes to make a 1‐µm‐thick, 10‐mm‐sized flat infrared detection chip, and also developed a perforation alignment process for precise alignment of the chiral meta‐mirror array to the detector pixel array. Based on the ingenious design and the advanced fabrication processes, the LWIR DoFPA circular polarimeter exhibits a high sensitivity to changes in both light intensity and Stokes parameter *S*_3_ within the spectral range of 10 µm‐11 µm. With this device, we demonstrate circular polarimetric imaging with the background of randomly polarized light been suppressed. To overcome the narrow‐band problem, we propose a multi‐resonance chiral meta‐mirror structure, and numerically demonstrate that the multi‐resonance metamaterial integrated QWIP with a spectral range being doubled. This work opens up a new avenue for the advancement of high‐performance LWIR DoFPA circular polarimeters.

## 6 Experimental Section

### Device Fabrication

The FPA chip was fabricated beginning with mesa etching of discrete pixels, followed by deposition of metal electrodes, under‐bump metallization (UBM), and a SiN_x_ passivation layer. The quantum well material was then connected to the ROIC via flip‐chip bonding, with epoxy resin reinforcement applied thereafter. The substrate is thinned to ≈200 µm by mechanical grinding and chemical etching until reaching the Al_0.5_Ga_0.5_As etch‐stop layer, then further thinned to achieve a final 1‐µm‐thick QWIP chip. Alignment marks were pre‐patterned during the UBM preparation. Subsequently, the alignment marks were exposed on the chip frontside through lithography and etching processes. ZEP 520‐A photoresist was spin‐coated, and precise patterning was achieved using electron‐beam lithography (EBL) aligned with these marks. Finally, chiral meta‐mirrors were formed by electron‐beam evaporation of a 50 nm‐thick metal layer (3 nm Ti/47 nm Au) followed by lift‐off processing. More details on the device fabrication can be found in Note S1 (Supporting Information).

The ROIC used in this work is a custom design developed by our research group, with architectural reference to the FLIR ISC9705 platform. This ROIC features direct charge‐integration architecture without signal amplification stages (gain = 1), exhibits readout noise below 1000 electrons under standard operating conditions, and provides a full well capacity of 18 million electrons.

### Optical Characterization

During testing, an achromatic quarter‐wave plate (G&H, Model XCN13, SN 1065) was utilized. The polarization mask employed in imaging experiments was fabricated using a stepper lithography system. The optical setups were schematically shown and described in detail in Note S5 and S10 (Supporting Information).

## Conflict of Interest

The authors declare no conflict of interest.

## Supporting information



Supporting Information

## Data Availability

The data that support the findings of this study are available from the corresponding author upon reasonable request.
