# Supplementary material for: Circular Polarimetric Imaging with a Metamaterial Integrated Long‐Wavelength Infrared Focal Plane Array
Source: Adv Sci (Weinh). 2025 Jul 25;12(39):e09292. doi: 10.1002/advs.202509292 (PMC12533386; doi:10.1002/advs.202509292)
Supplement: Supplementary file 1 — Supporting Information [file ADVS-12-e09292-s001.docx]

Supporting Information

**Circular polarimetric imaging with a metamaterial integrated long-wavelength infrared focal plane array**

*Tianyun Zhu^†^, Ling Wang^†^, Wenji Jing, Jie Deng, Jiexian Ye, Yujie Zhang, Zeshi Chu, Jing Zhou*, Xiaoshuang Chen*, Xiangyang Li, Wei Lu and Xuechu Shen*

1. Fabrication process of a metamaterial integrated QWIP FPA
2. Optoelectronic polarization eigenvectors acquisition in experiment
3. Optical properties of quantum well materials
4. The impact of the 3nm-thick titanium on simulation results
5. The optical setup for imaging and circular polarization spectral measurement
6. Optoelectronic properties of a 45° edge facet coupled QWIP
7. Origin of circular polarization selectivity in the chiral metamaterial integrated structure
8. Performance metrics of the QWIP FPA at varying operating temperatures
9. The calculation of noise equivalent *S*_3_ difference (NE*S*_3_D)
10. The design and optical characteristics of the imaging mask
11. Spectral broadening enabled by multimodal optical resonances
12. Fabrication process of a metamaterial integrated QWIP FPA

Prior to the integration of the differential chiral metamaterial, discrete pixel mesas are first created through etching. Subsequently, metal electrodes, under-bump metallization (UBM), and a SiN_x_ passivation layer are fabricated. Flip-chip bonding is then employed to establish a connection between the quantum well material and the readout circuit. Epoxy resin is used to fill the channels and improve the mechanical strength of the chip after curing. Then, the substrate is thinned to approximately 200 μm via mechanical grinding, followed by chemical etching using a solution of citric acid: hydrogen peroxide = 4 : 1 (volume ratio) until the Al_0.5_Ga_0.5_As etch-stop layer is exposed. Finally, the QW substrate is thinned by approximately 500 μm, resulting in an QWIP chip with a final thickness of about 1 μm, a lateral size of 10 mm × 8 mm, and a smooth surface.

The alignment marks are fabricated concurrently during the UBM preparation. These original alignment marks are covered by the chip after flip-chip bonding process. Our alignment procedure consists of three key steps: First, we locate the approximate positions of the alignment marks (with an alignment accuracy of 0.5 μm) using reference markers on the readout circuit from the flip-chip bonding process. Next, we expose the alignment marks completely by opening a window for each mark through lithography and etching processes. Finally, we perform electron-beam lithography with these exposed alignment marks to create metamaterial patterns on the top of the chip with an alignment accuracy of 20 nm. After that, a lift-off process is conducted to realize the 50 nm-thick metamaterial (3 nm Ti/47 nm Au).


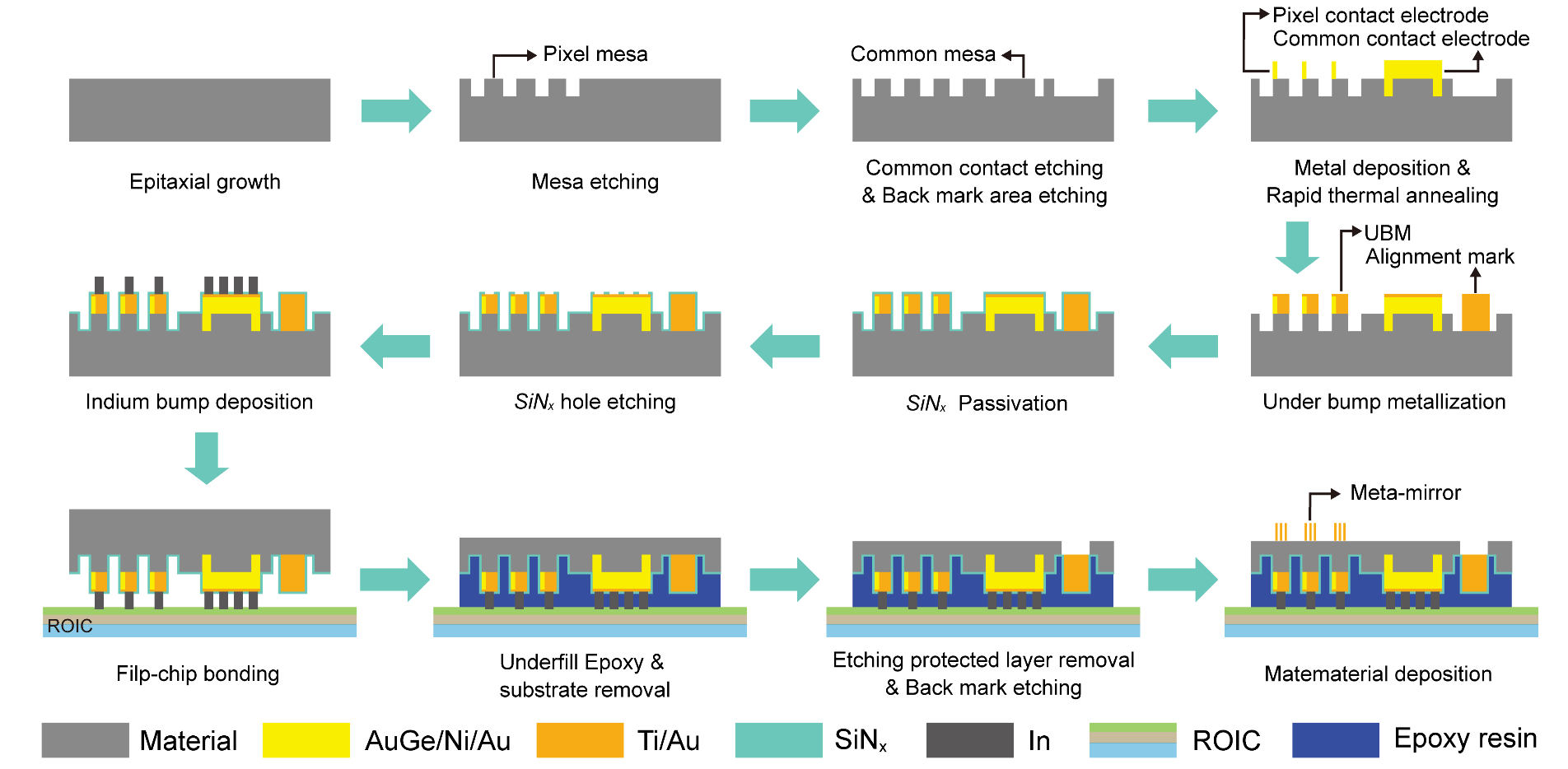


**Figure S1****.** Fabrication process of a QWIP focal plane array.

1. Optoelectronic polarization eigenvectors acquisition in experiment

The acquisition of photoelectric polarization feature vectors (OPEVs) can be achieved by performing at least four photocurrent measurements corresponding to four linearly independent incident polarization states.^[1]^ The OPEV is represented as$\left[\begin{matrix} \mu_{0} & \mu_{1} & \mu_{2} & \mu_{3} \end{matrix}\right]$, and the four measured photocurrent values are denoted as$\left[\begin{matrix} J_{0} & J_{1} & J_{2} & J_{3} \end{matrix}\right]$, The relationship between the photocurrents and the OPEV can be expressed as:

$\left[\begin{matrix} J_{0} \\ J_{1} \\ J_{2} \\ J_{3} \end{matrix}\right]=\left[\begin{matrix} S_{00} & S_{01} & S_{02} & S_{03} \\ S_{10} & S_{11} & S_{12} & S_{13} \\ S_{20} & S_{21} & S_{22} & S_{23} \\ S_{30} & S_{31} & S_{32} & S_{33} \end{matrix}\right]\left[\begin{matrix} \mu_{0} \\ \mu_{1} \\ \mu_{2} \\ \mu_{3} \end{matrix}\right]$ (S1)

Therefore, as long as the photocurrents for four different incident polarization states are known, the values of the OPEV can be obtained. However, using more redundant test Stokes vectors will enhance the accuracy of solving for the OPEV. Thus, here we use a series of photocurrent values obtained from rotating the quarter-wave plate as shown in **Figure.2 f** of the main text to fit and obtain the OPEV of the QWIP integrated with the left-handed chiral meta-mirror. In this setup, the half-wave plate is fixed at a constant angle, while the angle of the quarter-wave plate (the angle α between the fast axis of the quarter-wave plate and the transmission axis of linear polarizer) is rotated. The relationship between the photocurrent and α can be expressed as:

$J\left(\alpha\right)=\left[\mu_{0}+\frac{1}{2}\mu_{1}\right]+\left[\frac{1}{2}\mu_{1}\cos\left(4\alpha\right)\right]+\left[\frac{1}{2}\mu_{2}\sin\left(4\alpha\right)\right]+\left[-\mu_{3}\sin\left(2\alpha\right)\right]$ (S2)

For a super pixel with circular polarization differential functionality, avoiding the influence of linear polarization is crucial. Due to the mirror symmetry of the chiral meta-mirrors, the two subpixels respond identically to the randomly polarized, horizontally polarized, or vertically polarized linearly light, which cancels out to zero upon subtraction, resulting in no photoresponse for S_0_ and S_1_. Then, the OPEV of the sub-pixel is evaluated to be $\left[\begin{matrix} 0 & 0 & 2\mu_{2} & 2\mu_{3} \end{matrix}\right]$. The fitted μ_2_ and μ_3_ are presented in **Figure S2**, with μ_3_ approaching 1 and μ_2_ remaining close to 0. At the wavelength of 10.5 μm, where μ_2_ = −0.20 A/W and μ_3_ = 1.65 A/W, approximately 90% of the energy of the final signal is contributed by the circularly polarized component, while the remaining 10% stems from 45° or 135° linearly polarized light, making the Stokes parameter S_2_ negligible in the final output signal. Therefore, the signal from the checkerboard FPA integrated with different chiral meta-mirrors is almost proportional to S_3_, allowing for near-in-situ measurement of the Stokes parameter S_3_.


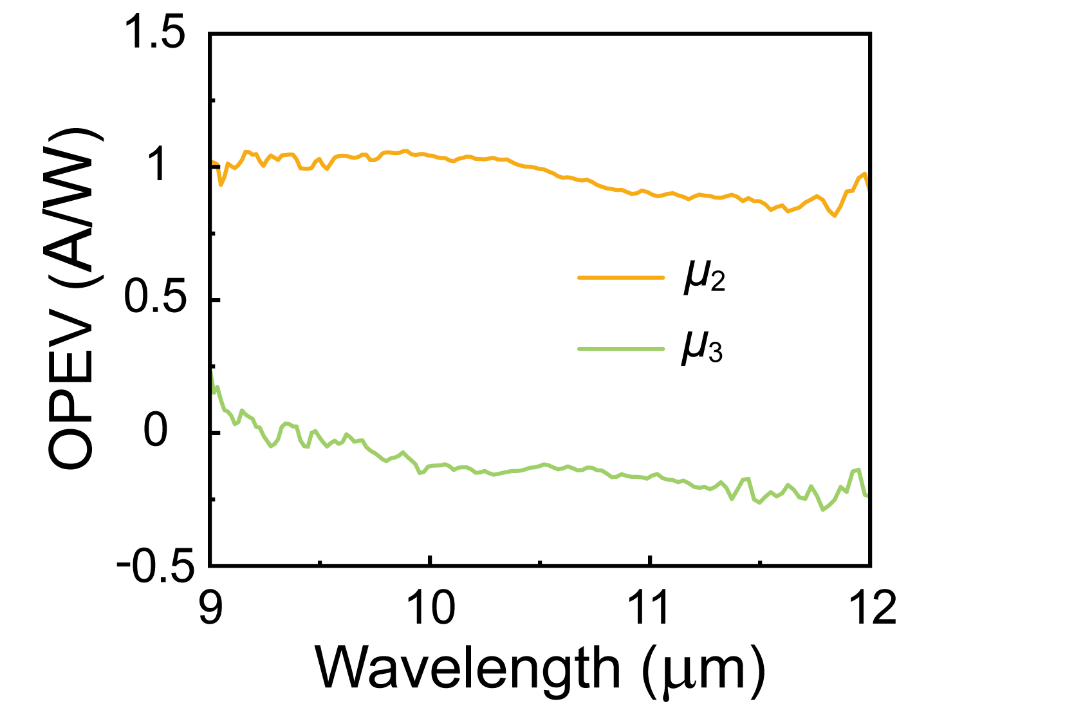


**Figure S2.** Optoelectronic polarization eigenvectors acquisition in experiment.

1. Optical properties of the quantum well material

Due to the selection rule of the intersubband transition, the quantum wells (QWs) can only absorb the light with a *z*-component electric field.^[2-3]^ Therefore, during simulation the QWs can be regarded as a uniaxial medium with a diagonal dielectric constant tensor: *ε* = diag (*ε_x_*, *ε_y_*, *ε_z_*). Along the x- or y-direction, the medium is a dielectric with the relative permittivity *ε_x_* = *ε_y_* = *ε_GaAs_*. In the z-direction, the intersubband transition is described by a Lorentz oscillator^[4]^:

$\varepsilon_{z}=\varepsilon_{GaAs}+\frac{\varepsilon_{GaAs}f_{12}\omega_{p}^{2}}{\omega_{0}^{2}-\omega^{2}-i\gamma\omega}$. (S3)

where f_12_ = 0.857, ω_p_ = 24.8 meV, ω_0_ = 120.6 meV, and *γ* = 21 meV. All the parameters of the Lorentz oscillator model are determined by fitting the model to the photocurrent spectrum of a 45° edge facet coupled QWIP made of the same QWs materials. More information about the 45° edge facet coupled QWIP can be found in Note S5. **Figure S3** shows the spectra of the real and imaginary parts of the relative permittivity of QWs.


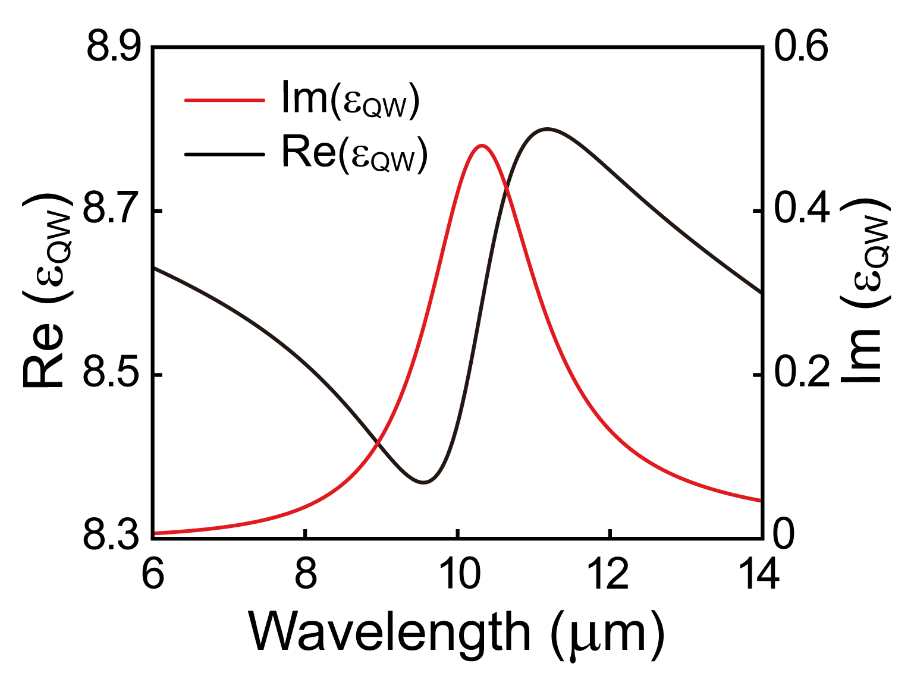


**Figure S3.** Real and imaginary parts of the permittivity of QWs along the *z*-direction.

1. The impact of the 3nm-thick titanium on simulation results

In the main text, the Z-antenna layer is assumed to be made of gold (Au). However, in real experiment, the Z-antenna layer consists of a 3-nm-thick titanium (Ti) layer for adhesion and with a 47nm-thick gold layer. To ensure simulation accuracy, we conducted additional modeling by incorporating the 3-nm-thick Ti layer into the structure, as depicted in **Figure S4a**. **Figure S4b** exhibits the spectra of reflection (R), QW absorptance (A_QW_), and metal absorptance (A_metal_) under either LCP or RCP illumination. Notably, the Ti layer slightly reduces the peak QW absorptance from 26.7% to 25.0% under LCP illumination, and reduces the QW absorptance from 0.63% to 0.60% under RCP illumination. Crucially, the peak CPER of the model with the 3-nm Ti layer reaches 48.4 (**Figure S4c**), which is slightly higher than that of the model without the 3-nm Ti layer. This confirms that the Ti layer does not compromise the polarization discrimination capability.


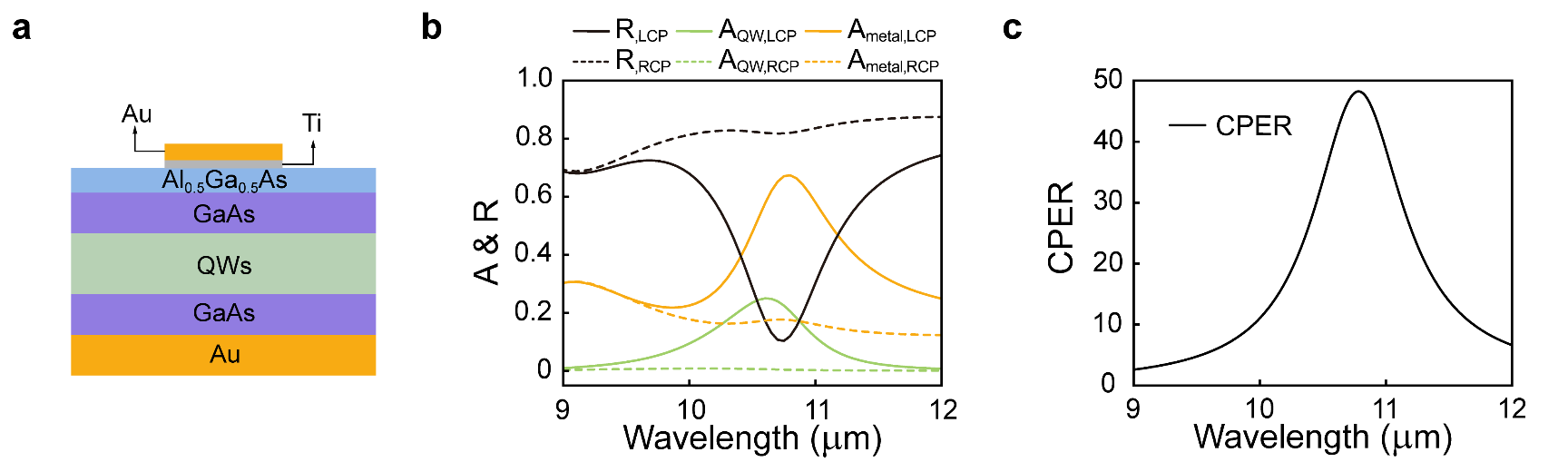


**Figure S4.** a) Schematic of the *xz* cross-section of the left-handed chiral meta-mirror integrated QWIP with a 3-nm Ti layer. b) Simulated spectra of reflectance (R), QW absorptance (A_QW_), and metal absorptance (A_metal_) of the QWIP with a 3-nm Ti layer under LCP and RCP illumination. c) CPER spectrum of the chiral meta-mirror integrated QWIP with a 3-nm Ti layer.

1. Optical setup for circular polarimetric imaging and circular polarization dependent photocurrent spectrum measurement

**Figure S5a** shows the optical setup for measuring the circular polarization dependent photocurrent spectrum of the sample device. The excitation light is the modulated infrared emission from the Fourier Transform Infrared Spectrometer (FTIR). The polarization state of the infrared light is controlled by a linear polarizer (LP) and a quarter-wave plate (QWP) before it arrives at the sample device. The transmission axis of the LP was fixed in the horizontal direction, converting the transmitted light into horizontally linearly polarized light. The fast axis of the QWP was then rotated to maintain a 45° angle with respect to the positive horizontal direction, generating left-handed circularly polarized (LCP) light. Further rotation of the QWP to a 135° angle relative to the horizontal direction produced right-handed circularly polarized (RCP) light. The photodetector of the FTIR system was replaced with our device to measure the photocurrent spectra under LCP and RCP illumination, thereby determining the circular polarization extinction ratio (CPER) of the device.

**Figure S5b** illustrates the optical setup for single-pixel device scanning imaging. A laser source generates a 10.5 μm laser beam, which is converted into horizontally linearly polarized light by passing through a LP. After passing through a quarter-wave plate oriented such that its fast axis forms a 45° angle with the linear polarization direction, the incident light is transformed into LCP light. This light then passes through a mask before reaching the single-pixel device. The mask is a perforated metal plate with a specific pattern, where white areas are open and allow the incident light to pass through without altering its polarization state, whereas grey areas are metallic and block all incident light. The mask is mounted on a stepper motor that allows movement in the *x-y* plane, enabling the generation of a two-dimensional image in conjunction with the signal acquisition from the single-pixel device. Rotating the quarter-wave plate by 90° switches the incident light from LCP to RCP.


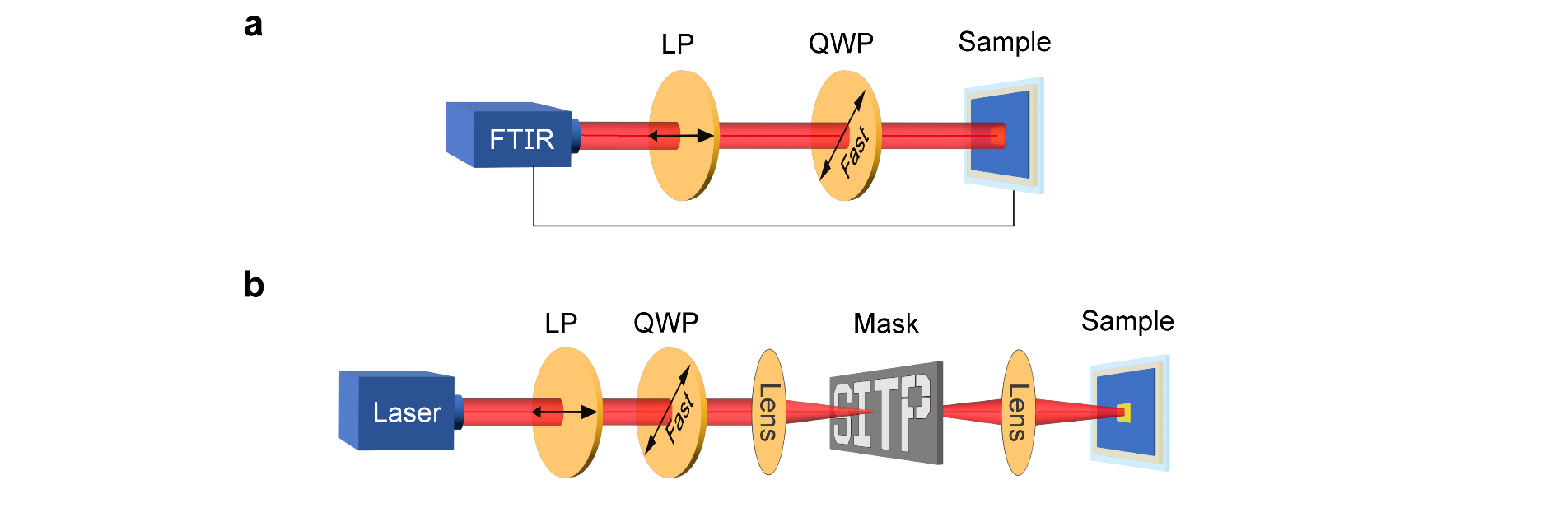


**Figure S5.** Schematic of the optical setup for QWIP performance measurement. a) Schematic diagram of the optical path of a single-pixel device for photocurrent spectrum measurement with the transmission axis of linear polarizer (LP) and the fast axis direction of quarter-wave plate (QWP) indicated by black arrows. The angles of 45° and 135°between them can respectively generate left-handed and right-handed circularly polarized light. b) Schematic diagram of the optical path of a single-pixel device for scanning imaging.

1. Optoelectronic properties of a 45° edge facet coupled QWIP

The 45° edge facet coupled quantum well infrared photodetector (QWIP) serves as a standard device to evaluate the optoelectronic properties of the QWs. As shown in **Figure S6a**, when the incident light illuminates the 45° edge facet of this device, a considerable z-component electric field (*E_z_*) is present in the QWs and it can excite the intersubband transition. Since there is no resonant light coupling, the photoresponse of this device stems solely from the intrinsic absorption of the QWs. Consequently, the photocurrent spectrum of the 45° edge facet coupled QWIP (**Figure S6b**) can be utilized to calibrate the dielectric constant of the QWs. As shown in **Figure** **2h**, this device achieves a peak responsivity of 0.40 A/W at a wavelength of 10.27 μm, and a responsivity of 0.38 A/W at 10.5 μm under LCP illumination. All these results were obtained at a bias voltage of −1.6 V and at an operating temperature of 10 K.


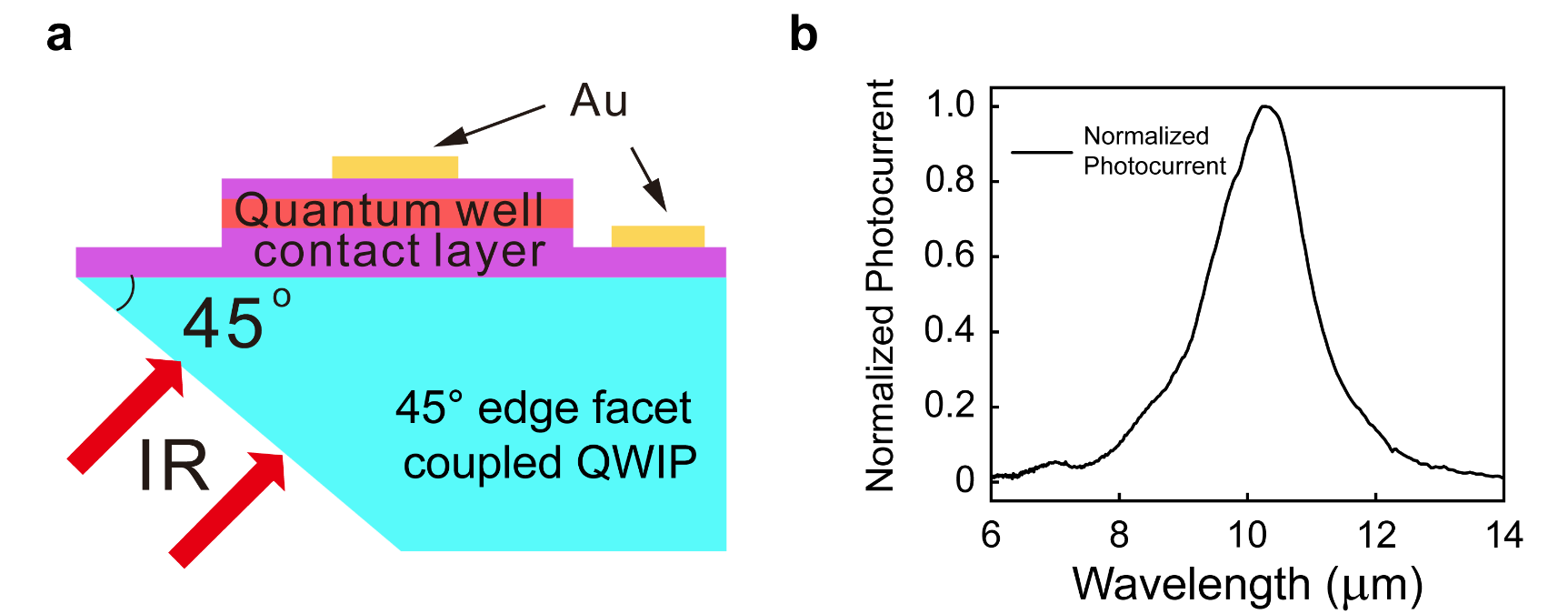


**Figure S6.** Optoelectronic properties of the 45° edge facet coupled QWIP. a) Schematic diagram of the 45° edge facet coupled QWIP. b) Normalized photocurrent spectrum.

1. Origin of circular polarization selectivity in the chiral metamaterial integrated structure

The circular polarization selectivity provided by the chiral metamaterial can be analyzed through the interference of the polarization-unconverted field and the polarization-converted field in the reflection.^[4-5]^ The field of circularly polarized light can be decomposed into two orthogonal vectors with a π/2 phase shift (e.g. *E_x_* and *E_y_*). The reflection of linearly polarized light (*E_x_* or *E_y_*) on the chiral metamaterial consists of the polarization-unconverted field (*r_xx_E_x_* or *r_yy_E_y_*) and the polarization-converted field (*r_xy_E_y_* or *r_yx_E_x_*). *r_xx_* and *r_yy_* denote the principal polarization reflection coefficients, while *r_xy_* and *r_yx_* represent the cross-polarization reflection coefficients. The reflected light can be calculated using a Jones matrix as follows:

$\left(\begin{matrix} E_{x}^{r} \\ E_{y}^{r} \end{matrix}\right)=\left(\begin{matrix} r_{xx} & r_{xy} \\ r_{yx} & r_{yy} \end{matrix}\right)\left(\begin{matrix} E_{x} \\ E_{y} \end{matrix}\right)$ (S4)

where $E_{x}^{r}$ and $E_{y}^{r}$ are the *x* and *y* components of the reflected electric field, respectively. The amplitude and phase of the reflected light are shown in **Figure S7a, b**. The interference between the unconverted field and the converted field for LCP and RCP light is illustrated through vector calculations. For LCP incidence, the composite structure is observed to excite an effective resonance at 10.6 μm, which manifests as a distinct dip in the reflection spectrum. The unconverted field *r_xx_E_x_* (*r_yy_E_y_*) interferes with the converted field *r_xy_E_y_* (*r_yx_E_x_*) in a destructive manner, thereby reducing the reflected fields *E_x_*_,sum_ and *E_y_*_,sum_ (**Figure S7c, d**). Consequently, the incident light is effectively coupled into the QWs material. In contrast, for RCP incidence, the unconverted field *r_xx_E_x_* (*r_yy_E_y_*) constructively interferes with the converted field *r_xy_E_y_* (*r_yx_E_x_*), enhancing the reflected fields *E_x_*_,sum_ and *E_y_*_,sum_ (**Figure S7e, f**). This results in a fact that the majority of the incident light is reflected and unable to be coupled into the active material.


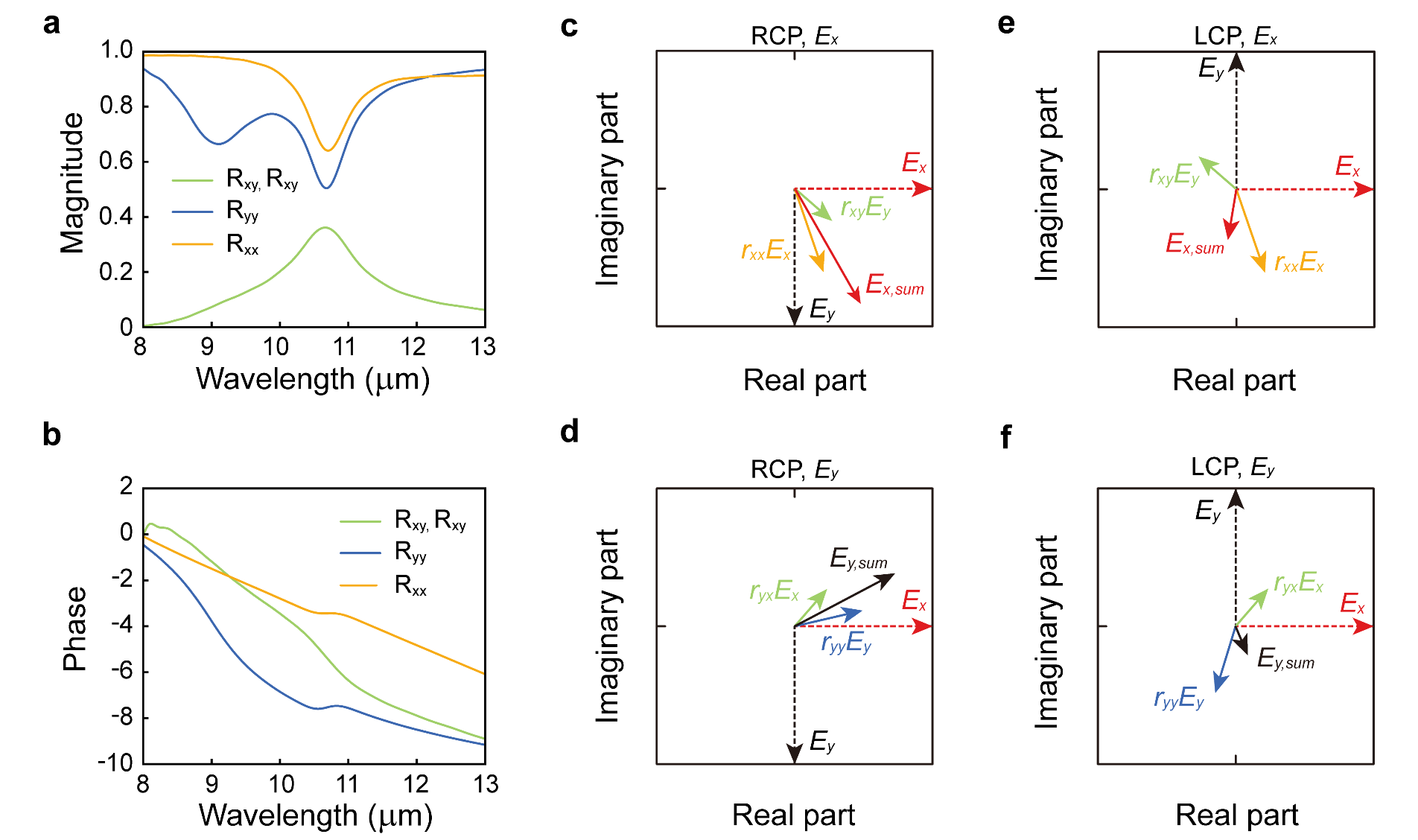


**Figure S7.** Analysis of the interference between the light reflected by the chiral meta-mirror integrated QWs. a, b) Magnitude (a) and phase (b) of the reflection coefficients for *E_x_* and *E_y_*. c-f) Vector plots illustrating destructive (c, d) and constructive (e, f) interference between the unconverted reflected field, *r_xx_E_x_* (or *r_yy_E_y_*) and the converted reflected field *r_xy_E_y_* (or *r_yx_E_x_*) under LCP (c, d) and RCP illumination (e, f) at the wavelength of 10.6 μm.

1. The calculation of noise equivalent *S*_3_ difference (NE*S*_3_D)

For the super-pixel device, the Stokes parameter *S*_3_ is proportional to the response of the device (*R*_s_):

$R_{s}=\left[\begin{matrix} S_{0} & S_{1} & S_{2} & S_{3} \end{matrix}\right]\left[\begin{matrix} 0 \\ 0 \\ 0 \\ 2\mu_{3} \end{matrix}\right]=2S_{3}\cdot\mu_{3}$ (S5)

Therefore, under a fixed incident light power *P*_0_, the change in the Stokes parameter *S*_3_ (Δ*S*_3_) is proportional to the change in the response of the device (Δ*R*_s_) (or the change in the signal voltage Δ*V*_s_).

$\Delta S_{3}=\frac{\Delta R_{\text{s}}}{2\mu_{3}}=\frac{\Delta R_{\text{s}}\cdot P_{0}}{2\mu_{3}\cdot P_{0}}=\frac{\Delta V_{\text{s}}}{2\mu_{3}\cdot P_{0}}$ (S6)

Therefore, when Δ*V*_s_ = *V*_n_ (*V*_n_ represents the noise voltage of individual pixels under 293 K background thermal radiation), the system reaches its minimum detectable change in the Stokes parameter *S*_3_, which is defined as the noise equivalent *S*_3_ difference (NE*S*_3_D):

$\text{NE}S_{3}\text{D}=\frac{V_{\text{n}}}{2\mu_{3}\cdot P}$ (S7)

As shown in Figure 4c of the main text, we computed the room temperature (293 K) background limited NE*S*_3_D, and the mean value obtained is 1.16 × 10^−4^. In Formula 7, *P* represents the optical power caused by a room temperature background of 293 K.

The noise voltage *V*_n_ is calculated as:

$V_{n}\left(i,j\right)=\frac{1}{K}\sqrt{\frac{1}{F-1}\sum_{f=1}^{F} \left\{\bar{V}_{s}\left(i,j\right)-V_{s}\left[(i,j),f\right]\right\}^{2}}$ (S8)

where *V*_n_ (*i*, *j*) represents the noise voltage of the super pixel located at column *i* and row *j*. The signal voltage $V_{s}\left[(i,j),f\right]$ corresponds to the *f*-th (where *f* = 1 to 100) frame in the two-dimensional array data measured at 293 K. $\bar{V}_{s}\left(i,j\right)$ denotes the average signal voltage across all *F* = 100 frames for the same super pixel.

1. Performance metrics of the QWIP FPA at varying operating temperatures

We characterized the noise equivalent temperature difference (NETD) and detectivity (*D*^*^) of our device across an operating temperature range from 20 K to 60 K. At 45 K, the device achieved a minimum NETD of 32 mK (**Figure S8a**). With the increasing operating temperature, thermal noise escalates, resulting in an NETD of 48.2 mK at 60 K. However, decreasing the temperature to 40 K yields an NETD of 33.5 mK, while temperatures below 40 K exhibit rapid noise degradation—reaching over 2500 mK at 20 K. This behavior originates from the restricted operational temperature range (40-100 K) of our readout integrated circuit (ROIC), where sub-40 K conditions induce excessive circuit noise due to operational instability. As shown in **Figure S8a**, *D*^*^ follows an identical trend, peaking at 4.26 × 10^10^ Jones at 45 K, with both higher and lower temperatures degrading performance. Consequently, 45 K was selected as the optimal operating temperature for reporting device metrics.


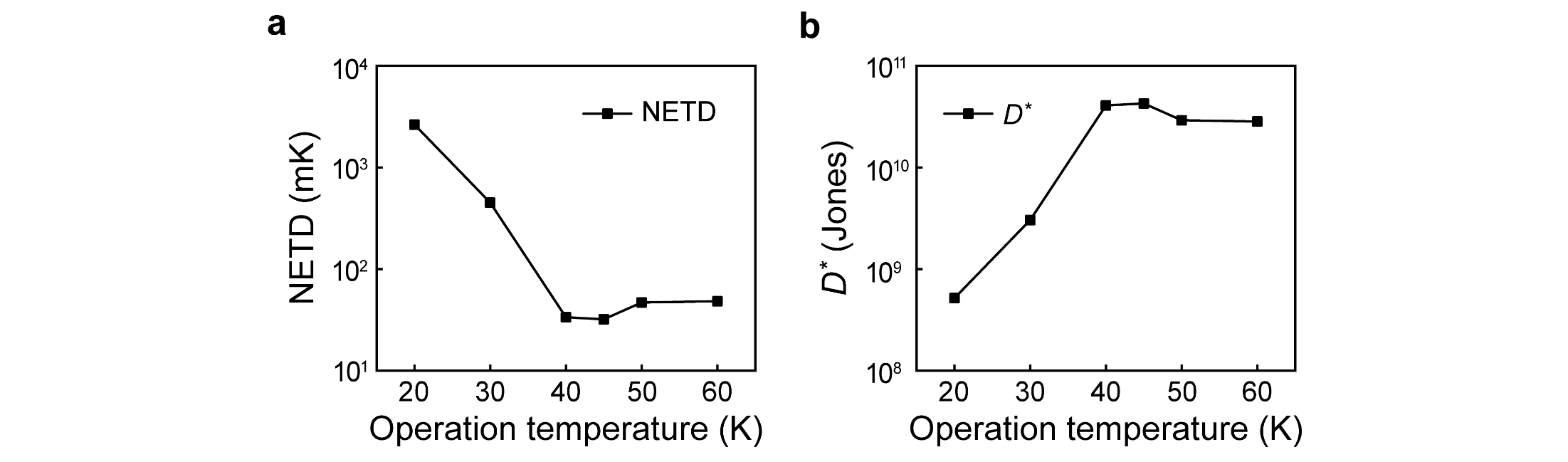


**Figure S8.** a) Operating temperature-dependent NETD averaged over all the pixels. b) Operating temperature-dependent detectivity (*D*^*^) averaged over all the pixels.

1. The design and optical characteristics of the imaging mask

To create a target with three polarization states—left-circularly polarized (LCP), right-circularly polarized (RCP), and randomly polarized—we fabricated the imaging mask shown in **Figure S9a**. The mask substrate is a high-resistance silicon wafer that has undergone double-side polishing and exhibits high transmission in the long-wave infrared (LWIR) range. Using a stepper lithography, we prepared three different micro-nano structures in distinct regions of the Si wafer to generate and pattern specific polarization states of light, as illustrated in **Figure S9b**. The surface-emitting blackbody produces randomly polarized light, which, after passing through the imaging mask, becomes x-polarized light, *y*-polarized light, and randomly polarized light in the three respective regions. By adding a quarter-wave plate after the mask and controlling the angle of its fast axis, the *x*-polarized light (Region I) and *y*-polarized light (Region II) can be converted to LCP and RCP, respectively, while the polarization state of the randomly polarized light remains unchanged. Further rotating the quarter-wave plate by 90° swaps the circular polarization states of Regions I and II, leaving Region III unaffected.

To achieve special polarization states in the transmitted light, Regions I, II, and III were specifically designed with longitudinal gratings, transverse gratings, and a circular polarization-insensitive structure, respectively. Scanning electron microscope (SEM) images of these structures are shown in **Figure S9d-f**. Both the transverse and longitudinal gratings share the same structural parameters, which, due to deep sub-wavelength scale limitations, significantly suppress the transmission of light polarized parallel to the grating direction, achieving a high linear polarization extinction ratio across the LWIR band, as shown in **Figure S9g**, thus ensuring the purity of the emitted polarization. Region III, designed as a circular structure that does not affect the polarization characteristics of the transmitted light, has a higher transmittance than the other two regions. This design mimics scenarios where the intensity of circularly polarized signals is lower than that of the randomly polarized background noise, highlighting the detection of circular polarization states while avoiding interference from intensity information, thereby creating more stringent test conditions for our circular polarization imaging.


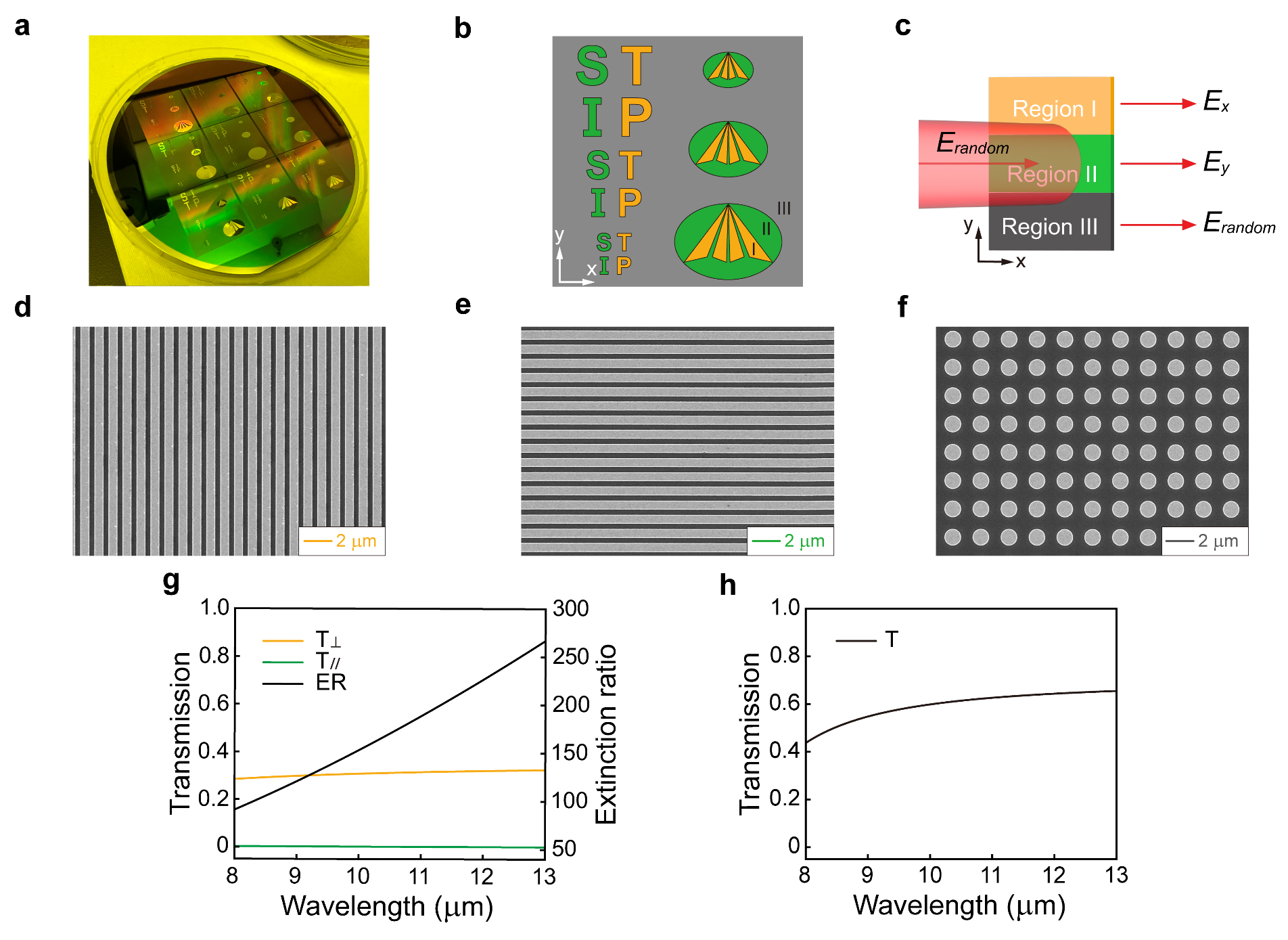


**Figure S9.** Design and functional implementation of the imaging mask. a) Photograph of the imaging mask. b) Schematic diagram of imaging mask, with three colors representing regions composed of three different nanostructures. c) Functional diagram of the mask in Figure. b, where randomly polarized light becomes *x*-polarized after passing through region Ⅰ, y-polarized after passing through region Ⅱ, and randomly polarized after passing through region Ⅲ. d-f) SEM images of nanostructures in regions Ⅰ, Ⅱ, and Ⅲ. g) Transmission of randomly polarized light after passing through region Ⅰ and region Ⅱ for polarization perpendicular to the grating direction (T_⊥_) and parallel to the grating direction (T*_∥_*). Extinction ratio (ER = T_⊥_ / T*_∥_*) of the mask. h) Transmission of randomly polarized light after passing through region Ⅲ.

1. Spectral broadening enabled by multimodal optical resonances

The high circular polarization discrimination capability of our proposed approach relies on optical resonance effects, inherently restricting device operation to a narrow spectral range. However, broad-spectrum sensitivity with polarization discrimination is essential for many applications. To this end, we design a chiral meta-mirror with multiple resonances and numerically demonstrate that this new metamaterial could expand the spectral range from 1 μm to 2 μm. The new metamaterial is a combination of three chiral meta-mirror structures. Each of them consists of a Z-antenna top layer, a dielectric spacing layer and a bottom metal reflector. The periodicity in the *y*-direction (*P_y_*) of the Z-antenna determines the resonance wavelength of the SPP mode. Thus, combining three Z-antenna structures with different *P_y_* values results in a multi-resonance spectrum and hence an effective broad spectral range. The three Z-antenna structures share the same periodicity in the *x*-direction (*P_x_*). The new metamaterial has a periodicity of 3*P_x_* in the *x*-direction and a periodicity of the least common multiple of *P_y_*_1_, *P_y_*_2_, *P_y_*_3_ in *y*-direction. The Z-antenna top layer of the new metamaterial is depicted in **Figure S10a**, and the detailed structural parameters are as follows: *P_x_* = 2.5 μm, *P_y_*_1_ = 3.333 μm, *L*_1_ = 2.166 μm *W*_1_ = 0.431 μm, *B*_1_ = 0.783 μm, *P_y_*_2_ = 3.75 μm, *L*_2_ = 2.437 μm *W*_2_ = 0.431 μm, *B*_2_ = 0.783 μm, *P_y_*_3_ = 4 μm, *L*_3_ = 2.6 μm *W*_3_ = 0.431 μm, *B*_3_ = 0.783 μm. The simulated QW absorptance spectrum of the left-handed multi-resonance metamaterial integrated QWIP under LCP illumination (**Figure S10b**) shows that the peak absorptance (23.9% at 10.6 μm) is similar to that of the device presented in our manuscript, and the full width at half maximum (FWHM) (2 μm) is doubled. Crucially, the circular polarization extinction ratio (CPER) remains > 10 across 9.4-11.4 μm (**Figure S10c**), and achieves a peak value of 21.4 at 10.45 μm.


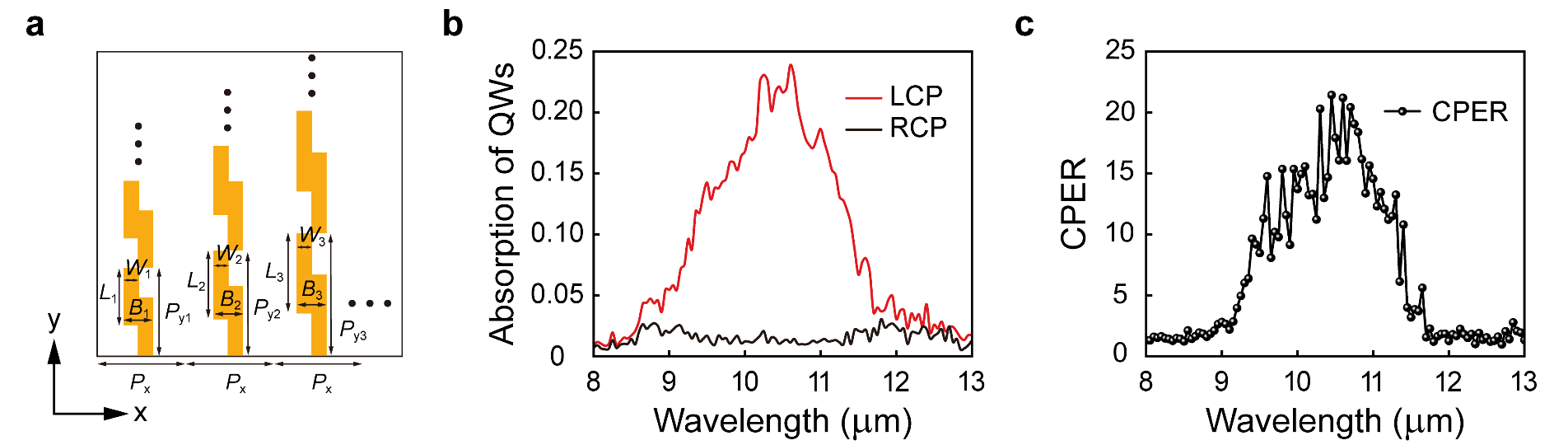


**Figure S10.** a) Schematic of the Z-antenna top layer of the mutli-resonance metamaterial. b) QW absorptance spectra of the multi-resonance metamaterial integrated QWIP under LCP and RCP illumination. c) CPER spectrum of the multi-resonance metamaterial integrated QWIP.

References

[1] J. Deng, M. Shi, X. Liu, et al., An on-chip full-Stokes polarimeter based on optoelectronic polarization eigenvectors. *Nature Electronics* **2024**, *7*, 1004–1014.

[2] B. F. Levine, Quantum-well infrared photodetectors. *Journal of Applied Physics* **1993**, *74* (8), R1-R81.

[3] H. T. Miyazaki, T. Mano, T. Kasaya, et al., Synchronously wired infrared antennas for resonant single-quantum-well photodetection up to room temperature. *Nat Commun* **2020**, *11* (1), 565.

[4] Z. Chu, J. Zhou, X. Dai, et al., Circular Polarization Discrimination Enhanced by Anisotropic Media. *Advanced Optical Materials* **2020**, *8* (9), 1901800.

[5] W. Li, Z. J. Coppens, L. V. Besteiro, et al., Circularly polarized light detection with hot electrons in chiral plasmonic metamaterials. *Nat Commun* **2015**, *6* (1), 8379.
